# Numerical solution of neutral delay differential equations using orthogonal neural network

**DOI:** 10.1038/s41598-023-30127-8

**Published:** 2023-02-23

**Authors:** Chavda Divyesh Vinodbhai, Shruti Dubey

**Affiliations:** Department of Mathematics, Indian Institute of Technology Madras, Chennai, Tamil Nadu 600036 India

**Keywords:** Applied mathematics, Computational science, Information technology

## Abstract

In this paper, an efficient orthogonal neural network (ONN) approach is introduced to solve the higher-order neutral delay differential equations (NDDEs) with variable coefficients and multiple delays. The method is implemented by replacing the hidden layer of the feed-forward neural network with the orthogonal polynomial-based functional expansion block, and the corresponding weights of the network are obtained using an extreme learning machine(ELM) approach. Starting with simple delay differential equations (DDEs), an interest has been shown in solving NDDEs and system of NDDEs. Interest is given to consistency and convergence analysis, and it is seen that the method can produce a uniform closed-form solution with an error of order $2^{-n}$, where *n* is the number of neurons. The developed neural network method is validated over various types of example problems(DDEs, NDDEs, and system of NDDEs) with four different types of special orthogonal polynomials.

## Introduction

Delay differential equation (DDE) plays a crucial role in epidemiology, population growth, and many mathematical modeling problems. In DDEs, the dependent variable depends not only on its current state but also on a specific past state. One type of DDE in which time delays are included in the state derivative is called the neutral delay differential equation (NDDE). Delay terms are classified into three types: discrete, continuous, and proportional delay. In this paper, we are focusing on proportional DDEs and NDDEs. One famous example of proportional delay differential equations is the pantograph differential equation which was first introduced in^1^.

Generally, the exact solution of delay differential equations is complicated to find, and due to the model’s complexity, many DDEs do not have an exact solution. Various numerical schemes have been developed over the years to find the approximate solution of delay differential equations. There are several articles^2–9^ that illustrate some exact and numerical methods for approximate solutions of DDEs and NDDEs.

Artificial neural networks(ANNs) have been utilised to produce an approximate solution of differential equations for the past 22 years. A neural network approach for several ordinary and partial differential equations was first proposed by Lagaris et al. in^10^. The approximate solution delivered by the artificial neural networks has a variety of advantages: (i) The derived approximation of the solution is in closed analytic form. (ii) The generalization ability of an approximation is excellent. (iii) Discretization of derivatives is not required. Many articles on approximation artificial neural network solutions to different differential equations are available in the literature^11–20^. As far as we know, the studies for obtaining an approximate solution to delay differential equations using artificial neural networks are limited. There is very little literature available for solving delay differential equations using ANNs. J. Fang et al. solved first-order delay differential equations with single delay using ANN^21^. In^22^, Chih-Chun Houe et al. obtained approximate solutions of proportional delay differential equation using ANN. All these artificial neural network approaches suffer from common problems: (1) All the algorithms are time-consuming and therefore they are computationally expansive numerical optimization algorithms, (2) They completely depend on the trial solution, which is difficult to construct for higher dimensional problems. Recently in^23^, Manoj and Shagun obtained an approximate solution of differential equations using an optimization-free neural network approach in which they trained the network weights using ELM algorithm^24^. In^25^, authors solved the first-order pantograph equation using the optimization-free ANN approach. Linear first-order delay differential-algebraic equations have been solved using Legendre neural network in^26^.

This work presents an orthogonal neural network with an extreme learning machine algorithm(ONN-ELM) to obtain an approximate solution for higher-order delay differential equations, neutral delay differential equations, and a system with multiple delays and variable coefficients. The ONN model is a particular functional link neural network(FLNN)^12,27–29^ case. It has the advantage of fast and very accurate learning. The entire procedure becomes much quicker than a traditional neural network because it removes the high-cost iteration procedure and trains the network weights using the Moore-Penrose generalized inverse. The following are the benefits of the proposed approach:

- It is a single hidden layer neural network, we only need to train the output layer weights by randomly selecting the input layer weights.
- We use an unsupervised extreme learning machine algorithm to train the output weights; no optimization technique is used in this procedure.
- It is simple to implement, accurate compared to other numerical schemes mentioned in the literature, and runs quickly.

This work considers four different orthogonal polynomials-based neural networks: (i) Legendre neural network, (ii) Hermite neural network, (iii) Laguerre neural network, and (iv) Chebyshev neural network with ELM for solving DDEs, NDDEs, and systems of NDDEs with multiple delays and variable coefficients. The interest is to find the orthogonal neural network among these four that can produce more accurate solution.

The layout of this paper is as follows. In “Preliminaries” section, we present some definitions and properties of orthogonal polynomials and a description of the considered problems. In “Orthogonal neural network” section, we describe the architecture of the orthogonal neural network(ONN) with an extreme learning algorithm(ELM). “Error analysis” section discusses the convergence analysis and error analysis. The methodology of the proposed method is presented in “Methodology” section. Various numerical illustrations are presented in “Numerical illustrations” section and a comparative study is given in “Comparative analysis” section.

## Preliminaries

In this section, first, we introduce basic definitions and some properties of the orthogonal polynomials. Throughout the paper, we will use $P_n(x)$ to represent the orthogonal polynomial of order *n*.

### Orthogonal polynomial

#### **Definition 1**

The orthogonal polynomials are special class of polynomials ${P_n(x)}$ defined on [*a*, *b*] that follow an orthogonality relation as,

$$\begin{aligned} \int _{a}^b g(x)P_m(x)P_n(x)dx = \delta _{m,n}k_n, \end{aligned}$$

where $n,m \in N$, $\delta _{m,n}$ is Kronecker delta, *g*(*x*) is a weight function and $k_{n} = \int _{a}^b g(x)[P_n(x)]^{2} dx$.

#### *Remark*

1. If a weight function $g(x)=1$, then the orthogonal polynomial $P_{n}(x)$ is called Legendre polynomial.
2. If a weight function $g(x)=(1-x^{2})^{-\frac{1}{2}}$, then the orthogonal polynomial $P_{n}(x)$ is called Chebyshev polynomial of first kind.
3. If a weight function $g(x)=e^{-x^{2}}$, then the orthogonal polynomial $P_{n}(x)$ is called Hermite polynomial.
4. If a weight function $g(x)=e^{-x}$, then the orthogonal polynomial $P_{n}(x)$ is called Laguerre polynomial.

#### Properties of orthogonal polynomials

The following are some of the remarkable properties of a set of orthogonal polynomials:

- Each polynomial $P_n(t)$ is orthogonal to any other polynomial of degree $<n$ in a set of orthogonal polynomials $\{P_0(t),\ldots ,P_n(t),\ldots ,\}$.
- Any set of orthogonal polynomials has a recurrence formula that connects any three consecutive polynomials in the sequence, i.e., the relation $P_{n+1}(t)=(a_nt+b_n)P_{n}(t)-c_nP_{n-1}(t)$ exists, with constants $a_n, b_n, c_n$ depending on *n*.
- The zeroes of orthogonal polynomials are real numbers.
- There is always a zero of orthogonal polynomial $P_{n+1}(t)$ between two zeroes of $P_{n}(t)$.

### Moore-Penrose generalized inverse

In this section, the Moore-Penrose generalized inverse is introduced.

There can be problems in obtaining the solution of a general linear system $Ax = y$, where *A* may be a singular matrix or may even not be square. The Moore-Penrose generalized inverse can be used to solve such difficulties. The term generalized inverse is sometimes referred to as a synonym of pseudoinverse. More precisely, we define the Moore-Penrose generalized inverse as follows:

#### **Definition 2**

^30^ A matrix *B* of order $n\times m$ is the Moore-Penrose generalized inverse of matrix *A* of order $m\times n$, if the following hold

$$\begin{aligned} ABA = A, \,\, BAB = B,\,\,(AB)^T = AB,\,\,(BA)^T = BA, \end{aligned}$$

where $A^T$ denotes the transpose of matrix *A*. The Moore-Penrose generalized inverse of matrix *A* is denoted by $A^\dagger$.

#### **Definition 3**

$x_0 \in \mathbb {R}^n$ is said to be a minimum norm least-squares solution of a general linear system $Ax = y$ if for any $y \in \mathbb {R}^m$

$$\begin{aligned} \Vert x_0 \Vert \le \Vert x \Vert , \forall x \in \{ x: \Vert Ax-y \Vert \le \Vert Az-y \Vert , \forall z \in \mathbb {R}^n\} \end{aligned}$$

where $\Vert . \Vert$ is the Euclidean norm.

In other words, if a solution $x_0$ has the smallest norm among all the least-squares solutions, it is considered to be a minimum norm least-squares solution of the general linear system $Ax = y$.

#### **Theorem 1**

^30^ *Let* *B* *be a matrix with a minimum norm least-squares solution to the linear equation* $Ax = y$. *Then* $B = A^\dagger$*, the Moore-Penrose generalized inverse of matrix* *A*, *is both required and sufficient*.

### Problem definition

In this subsection, we present the general form of the pantograph equation, higher order delay differential equation, higher order neutral delay differential equation, and the system of higher order delay differential equation with variable coefficients and multiple delays.

#### The generalized Pantograph equation

Pantograph type equation arises as a mathematical model in the study of the wave motion of the overhead supply line to an electric locomotive. The following equation gives the generalized form of a pantograph type equation with multiple delays:

1 $$\begin{aligned} z'(t)=a(t)z(t) + \sum _{i=1}^{k} b_{i}(t)z(q_{i}t) + \sum _{j=1}^{l} c_{j}(t)z'(q_{j}t)+g(t), \end{aligned}$$

with initial conditions

2 $$\begin{aligned} z(t_{0})=z_{0}, \end{aligned}$$

where *g*(*t*), *a*(*t*), $b_{i}(t)$ and $c_{i}(t)$ is continuous function, $0<q_i,q_{j}<1$ for some $k,l \in \mathbb {N}$ and $t\in [t_0,t_1]$ for some, $t_0,t_1\in \mathbb {R}$.

#### Higher order DDEs and NDDEs

- Consider the general form of Higher-order DDEs with multiple delay
  3 $$\begin{aligned} z^{k}(t)=f\left( t,z(t),...z^{k-1}(t),z(q_{1}t),...z(q_{n}t)\right) , \end{aligned}$$
  with initial conditions
  4 $$\begin{aligned} z(t_{0})=z_{0},z'(t_{0})=z_{1},\ldots ,z^{k-1}(t_{0})=z_{k-1}, \end{aligned}$$
  where $q_{i}'s \in (0,1)$ for $i=$1,...,n and $z^{k}$ denotes the *kth* derivative of *z*(*t*).
- Consider the general form of Higher-order NDDEs with multiple delay
  5 $$\begin{aligned} \begin{aligned} z^{k}(t)=f( t,&z(t),...z^{k-1}(t),z(q^{1}_{1}t),\ldots ,\\&z(q^{1}_{n_{1}}t),z'(q^{2}_{1}t),\ldots ,z'(q^{2}_{n_{2}}t),\ldots ,z^{k}(q^{k+1}_{1}t),\ldots ,z^{k}(q^{k+1}_{n_{k+1}}t)), \end{aligned} \end{aligned}$$
  with initial condition
  6 $$\begin{aligned} z(t_{0})=z_{0},z'(t_{0})=z_{1},\ldots ,z^{k-1}(t_{0})=z_{k-1}, \end{aligned}$$
  where all $q_{i}^{j}\in (0,1)$ for $j=1,..,k+1$, $i=1,\ldots ,n_j$, $n_j, k\in \mathbb {N}$ and $z^{k}$ denotes the *kth* derivative of *z*(*t*).

#### Higher order system of DDE

Consider the general form of higher order coupled neutral delay differential equation with multiple delays as:

7 $$\begin{aligned}{} & {} \begin{aligned} z_{1}^{k}(t)=f(&t,z_{1}(t),...z_{1}^{k-1}(t),z_{2}(t),...z_{2}^{k}(t),z_{1}(q^{1}_{1}t),\ldots ,z_{1}(q^{1}_{n_{1}}t),z_{2}(p^{1}_{1}t),\ldots ,\\ {}&z_{2}(p^{1}_{m_{1}}t),z'_{1}(q^{2}_{1}t),\ldots ,z'_{1}(q^{2}_{n_{2}}t),z'_{2}(p^{2}_{1}t),\ldots ,z'_{2}(p^{2}_{m_{2}}t),\ldots ,z_{1}^{k}(q^{k+1}_{1}t),\ldots ,\\ {}&z_{1}^{k}(q^{k+1}_{n_{k+1}}t),z_{2}^{k}(p^{k+1}_{1}t),\ldots ,z_{2}^{k}(p^{k+1}_{m_{k+1}}t)), \\ z_1(t_{0})=z_{0}^{1}&,\,\,\,z_{1}'(t_{0})=z_{1}^{1},\ldots ,\,z_{1}^{k-1}(t_{0})=z_{k-1}^{1}, \end{aligned} \end{aligned}$$

8 $$\begin{aligned}{} & {} \begin{aligned} z_{2}^{k}(t)=g(&t,z_{1}(t),...z_{1}^{k-1}(t),z_{2}(t),...z_{2}^{k}(t),z_{1}(r^{1}_{1}t),\ldots ,z_{1}(r^{1}_{l_{1}}t),z_{2}(s^{1}_{1}t),\ldots ,\\ {}&z_{2}(s^{1}_{h_{1}}t),z'_{1}(r^{2}_{1}t),\ldots ,z'_{1}(r^{2}_{l_{2}}t),z'_{2}(s^{2}_{1}t),\ldots ,z'_{2}(s^{2}_{h_{2}}t),\ldots ,z_{1}^{k}(r^{k+1}_{1}t),\ldots ,\\ {}&z_{1}^{k}(r^{k+1}_{l_{k+1}}t),z_{2}^{k}(s^{k+1}_{1}t),\ldots ,z_{2}^{k}(s^{k+1}_{h_{k+1}}t)), \\ z_2(t_{0})=z_{0}^{2}&,\,\,\,z_{2}'(t_{0})=z_{1}^{2},\ldots ,\,z_{2}^{k-1}(t_{0})=z_{k-1}^{2}, \end{aligned} \end{aligned}$$

where $n_j,m_j,l_j,h_j \in \mathbb {N}$ and all $q_{i_{1}}^{j},p_{i_{2}}^{j},r_{i_{3}}^{j},s_{i_{4}}^{j} \in (0,1)$ for $j=1,..,k+1$, $i_1=1,\ldots ,n_j$, $i_2=1,\ldots ,m_j$, $i_3=1,\ldots ,l_j$, $i_4=1,\ldots ,h_j$.

## Orthogonal neural network

In this section, we introduce the structure of a single-layered orthogonal neural network(ONN) model with an extreme learning machine(ELM) algorithm for training the network weights.

### Structure of orthogonal neural network (ONN)

**Figure 1:**
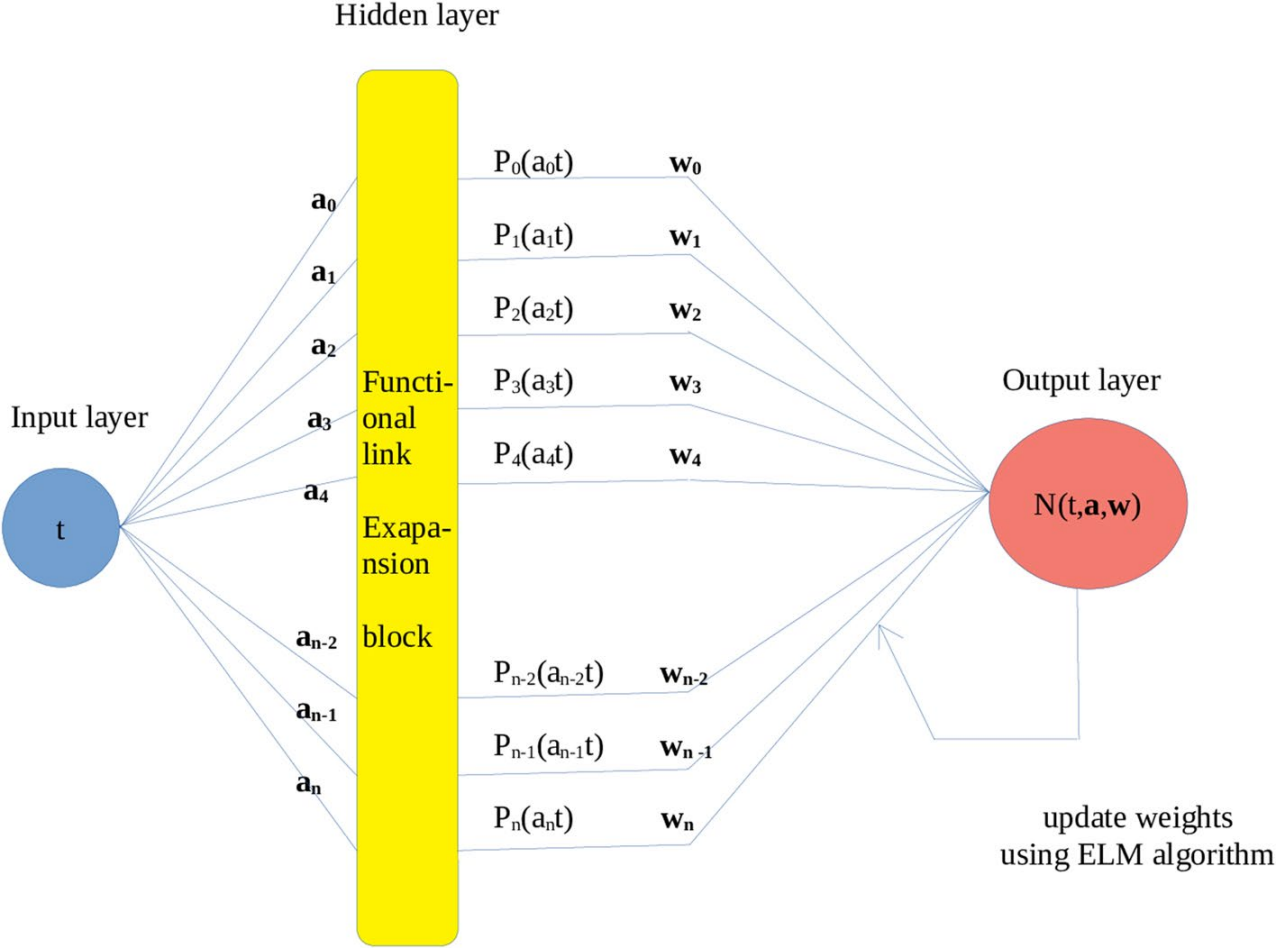
The structure of orthogonal neural network.

Orthogonal neural network(ONN) is a single-layered feed-forward neural network, which consists of one input neuron *t*, one output neuron $N(t,{\textbf {a}},{\textbf {w}})$ and a hidden layer is eliminated by the orthogonal functional expansion block. The architecture of an orthogonal neural network is depicted in Fig. 1.

Consider a 1-dimensional input neuron *t*. The enhanced pattern is obtained by orthogonal functional expansion block as follows:

$$\begin{aligned}{}[P_{0}(a_{0}t),P_{1}(a_{1}t),\ldots ,P_{n}(a_{n}t)]. \end{aligned}$$

Here $N(t,{\textbf {a}},{\textbf {w}})= \sum _{i=0}^{n} w_{i} P_{i}(a_{i}t)$ is the output of the orthogonal neural network, where $a_i's$ are randomly selected fixed weights and $w_{i}'s$ are the weights to be trained.

### Extreme learning machine (ELM) algorithm

For a given sample points $(t_j,y_j)$, $t_j \in \mathbb {R}^n$ and $y_j \in \mathbb {R}$, for $j=0,1,\ldots ,m$, a single-layer feed-forward neural network with $(n+1)$ neurons has the following output:

$$\begin{aligned} \sum _{i=0}^{n}w_ig_i(a_it_j), \,\,\, j=0,1,\ldots ,m, \end{aligned}$$

where $g_i$ is the activation function of *i*-th neuron in a hidden layer, $a_{i}'s$ are the randomly selected fixed weights between the input layer and hidden layer, and $w_{i}'s$ are the weights between the hidden layer and output, which need to be trained.

When the neural network completely approximates the given data, i.e., the output of the neural network and actual data are equal, the following relation hold:

9 $$\begin{aligned} \sum _{i=0}^{n}w_ig_i(a_it_j)=y_j.\,\,\,\, j=0,1,\ldots ,m. \end{aligned}$$

Equation (9) can be written in matrix form as:

10 $$\begin{aligned} A{\textbf {w}}={\textbf {b}}, \end{aligned}$$

where the hidden layer output matrix *A* is defined as follows:

11 $$\begin{aligned} A= \begin{pmatrix} g_{0}(a_0t_0)&{} g_{1}(a_1t_0) &{} \cdots &{} g_{n}(a_nt_0)\\ g_{0}(a_0t_1)&{} g_{1}(a_1t_1) &{} \cdots &{} g_{n}(a_nt_1)\\ \vdots &{} \vdots &{} \ddots &{} \vdots \\ g_{0}(a_0t_m)&{} g_{1}(a_1t_m) &{} \cdots &{} g_{n}(a_nt_m) \end{pmatrix}, \end{aligned}$$

and $${\textbf {w}}=[w_0,w_1,\ldots ,w_n]^T$$, $${\textbf {b}}=[y_0,y_1,\ldots ,y_m]^T$$.

For the given training points $t_{j}'s\in \mathbb {R}^n$ and the weights $a_{i}'s$, the matrix *A* can be calculated and the weights $w_{i}'s$ can be calculated by solving the linear system $A{\textbf {w}}={\textbf {b}}$.

#### **Theorem 2**

*The system* $\textbf{A}\textbf{w}=\textbf{b}$ *is solvable in the following several cases:*

1. *If* $\textbf{A}$ *is a square matrix, then* $\textbf{w}=\mathbf {A^{-1}}\textbf{b}$
2. *If* $\textbf{A}$ *is a rectangular matrix, then* $\textbf{w}=\mathbf {A^{+}}\textbf{b}$*, and* $\textbf{w}$ *is the minimal least square solution of* $\textbf{A}\textbf{w}=\textbf{b}$*. Here* $\mathbf {A^{+}}$ *is a pseudo inverse of* $\textbf{A}$.
3. *If* $\textbf{A}$ *is a singular matrix, then* $\textbf{w}=\mathbf {A^{+}}\textbf{b}$ *and* $\mathbf {A^{+}}$ *=* $\mathbf {A^{T}}(\lambda \textbf{I}+\textbf{A}\mathbf {A^{T}})^{-1}$*, where* $\lambda$ *is the regularization coefficient. We can set a value of* $\lambda$ *according to the specific instance.*

## Error analysis

This section will discuss the convergence result and error analysis of the ONN-ELM method for solving the delay and neutral delay differential equations.

### **Theorem 3**

^24^ *Let single layer feed-forward orthogonal neural network* $N(t,{\textbf {a}},{\textbf {w}})$ *be an approximate solution of one-dimensional neutral delay differential equation, for* $m+1$ *arbitrary distinct sample points* $(t_j,y_j)$ *for* $j=0,1,...m$*, where* $t_i,y_i \in \mathbb {R}$*, then the orthogonal expansion layer output matrix* *A* *is invertible, and* $\Vert A{\textbf {w}}-b \Vert =0$.

### **Theorem 4**

*Let* $z \in C^{\infty }(t_0,t_m)$, $\widehat{z}_{n}=N(t,{\textbf {a}},{\textbf {w}})$ *be the orthogonal neural network with* *n* *neurons in the hidden layer and* $e_{n}$ *be the absolute error with* *n* *hidden neurons, then* $\Vert e_{n}\Vert \rightarrow 0$ *as* $n\rightarrow \infty$.

### *Proof*

The Taylor expansion formula gives us the following expression for *z*(*t*) on $(t_0, t_m)$:

12 $$\begin{aligned} z(t)=z(t_0^+)+z'(t_0^+)(t-t_0)+\frac{z''(t_0^+)}{2!}(t-t_0)^2+...+\frac{z^{n}(c)}{n!}(t-t_0)^{n}, \,\,\, c\in (t_0,t_1). \end{aligned}$$

Let us define $z_n(t)=\sum _{i=0}^{n-1}\frac{z^i(t_0^+)}{i!}(t-t_0)^i$, then we get

13 $$\begin{aligned} \Vert z(t)-z_n(t) \Vert = \frac{1}{n!}\Vert z^{n}(c)(t-t_0)^{n} \Vert . \end{aligned}$$

Let $L=span\{P_0(t),P_1(t),\ldots ,P_n(t)\}$ and let $\widehat{z}_n(t)$ be the best approximation of *z*(*t*) in *L* given as, $\widehat{z}_n(t)=\sum _{i=0}^{n-1}w_iP_i(a_it)$, where $w_i$’s are the weights obtained by ELM algorithm. we get

14 $$\begin{aligned} \Vert z(t)-\widehat{z}_n(t) \Vert \le \Vert z(t)-\bar{z}(t) \Vert , \,\,\,\,\,\, \forall \bar{z}(t) \in L. \end{aligned}$$

In particular, taking $\bar{z}(t)=z_n(t)$ we have

15 $$\begin{aligned} \begin{aligned} \Vert e_n(t)\Vert&=\Vert z(t)-\widehat{z}_n(t)\Vert \\&\le \Vert z(t)-z_n(t) \Vert \\ {}&= \frac{1}{n!}\Vert z^{n}(c)(t-t_0)^{n} \Vert \end{aligned} \end{aligned}$$

Thus,

16 $$\begin{aligned} \begin{aligned} \Vert e_n(t)\Vert&\le \Vert \frac{z^n(c)}{n!}(t-t_0)^n\Vert \\ {}&\le \frac{M}{2^n}, \end{aligned} \end{aligned}$$

where, $$M=max\Vert z^{n}(c)(t-t_0)^n\Vert$$, for $$t\in (t_0,t_m)$$.

Moreover, from Eq. (16) we deduce that $\Vert e_n(t)\Vert \rightarrow 0$ for large value of *n*. This shows that ONN has high representational abilities and it can approximate the exact solution with almost no error. $\square$

## Methodology

This section explains the method to obtain an approximate solution of second-order NDDE using the ONN-ELM algorithm. It can be easily extended to the higher-order NDDE and the higher-order DDE is a special case of the higher-order NDDE.

Consider the general form of linear second-order NDDE

17 $$\begin{aligned} \begin{aligned} z''(t)&+a(t)z'(t)+b(t)z(t)+\sum _{j=1}^{m_1}c_{j}(t)z(\alpha _{j}t)+\sum _{k=1}^{m_2}d_{k}(t)z'(\beta _{k}t)\\ {}&+\sum _{l=1}^{m_3}e_{l}(t)z''(\gamma _{l}t)=f(t),\,\,\,\,\, t\in (a,b), \end{aligned} \end{aligned}$$

with initial condition $z(a)=z_0$ and $z'(a)=z_1$ or boundary condition $z(a)=z_2$ and $z(b)=z_3$, where $z_0,z_1,z_2,z_3 \in \mathbb {R}$, $a(t),b(t),c_{j}(t),d_{k}(t),e_{l}(t),f(t)$ are continuously differentiable function for $t \in (a,b)$ and $m_1,m_2,m_3 \in \mathbb {N}$.

Using ONN-ELM with *n* neurons, an approximate solution of Eq. (17) is obtained in the form:

18 $$\begin{aligned} \widehat{z}_{n}(t)=\sum _{i=0}^{n}w_{i}P_{i}(t), \end{aligned}$$

where $w_i$’s are the output weights that need to be trained and $P_i(t)$ is the *i*-th orthogonal polynomial.

Since the approximate solution obtained by the ONN-ELM algorithm is the linear combination of the orthogonal polynomials, it is infinitely differentiable and we have,

19 $$\begin{aligned} \widehat{z}'_n(t)= & {} \sum _{i=0}^{n}w_{i}P'_{i}(t), \end{aligned}$$

20 $$\begin{aligned} \widehat{z}''_n(t)= & {} \sum _{i=0}^{n}w_{i}P''_{i}(t), \end{aligned}$$

21 $$\begin{aligned} \sum _{j=1}^{m_1}\widehat{z}_n(\alpha _{j}t)= & {} \sum _{j=1}^{m_1}\sum _{i=0}^{n}w_{i}P_{i}(\alpha _{j}t), \end{aligned}$$

22 $$\begin{aligned} \sum _{k=1}^{m_2}\widehat{z}'_n(\beta _{k}t)= & {} \sum _{k=1}^{m_2}\sum _{i=0}^{n}\beta _{k}w_{i}P'_{i}(\beta _{k}t), \end{aligned}$$

23 $$\begin{aligned} \sum _{l=1}^{m_3}\widehat{z}''_n(\gamma _{l}t)= & {} \sum _{l=1}^{m_3}\sum _{i=0}^{n}\gamma _{l}^{2}w_{i}P''_{i}(\gamma _{l}t). \end{aligned}$$

Substituting Eqs. (18)–(23) into the second order neutral delay differential equation (17), we have

24 $$\begin{aligned} \begin{aligned}{}&\sum _{i=0}^{n}w_{i}P''_{i}(t)+a(t)\sum _{i=0}^{n}w_{i}P'_{i}(t)+b(t)\sum _{i=0}^{n}w_{i}P_{i}(t)+\sum _{i=0}^{n}w_{i}\sum _{j=1}^{m_1}c_{j}(t)P_{i}(\alpha _{j}t)\\ {}&+\sum _{i=0}^{n}w_{i}\sum _{k=1}^{m_2}\beta _{k}d_{k}(t)P'_{i}(\beta _{k}t)+\sum _{i=0}^{n}w_{i}\sum _{l=1}^{m_3}\gamma _{l}^{2}e_{l}(t)P''_{i}(\gamma _{l}t)=f(t). \end{aligned} \end{aligned}$$

We can write Eq. (24) as:

25 $$\begin{aligned} \sum _{i=0}^{n}w_{i} A_{i}(t)=f(t), \end{aligned}$$

where,

$$\begin{aligned} \begin{aligned} A_{i}=&P''_{i}(t)+a(t)P_{i}(t)+b(t)P_{i}(t)+\sum _{j=1}^{m_1}c_{j}(t)P_{i}(\alpha _{j}t)+ \sum _{k=1}^{m_2}\beta _{k}d_{k}(t)P'_{i}(\beta _{k}t)\\ {}&+\sum _{l=1}^{m_3}\gamma _{l}^{2}e_{l}(t)P''_{i}(\gamma _{l}t). \end{aligned} \end{aligned}$$

Using the discretization of interval [*a*, *b*] as $a=t_0<t_1<,\cdots ,<t_m=b$ for $m\in \mathbb {N}$, define $f_m=f(t_m)$. At these discretized points, Eq. (25) is to be satisfied, that is:

26 $$\begin{aligned} \sum _{i=0}^{n}w_iA_{i}(t_m)=f(t_m),\,\,\,\ \forall m \in \mathbb {N}. \end{aligned}$$

Equation (26) can be written as a system of equations as:

$$\begin{aligned} A_{1}{} {\textbf {w}}=b_1, \end{aligned}$$

where $${\textbf {w}}=[w_0,w_1,\ldots ,w_n]^{T}$$,

$$\begin{aligned} A_1= \begin{pmatrix} A_{0}(t_0) &{} A_{1}(t_0) &{} \cdots &{} A_{n}(t_0)\\ A_{0}(t_1) &{} A_{1}(t_1) &{} \cdots &{} A_{n}(t_1)\\ \vdots &{} \vdots &{} \ddots &{} \vdots \\ A_{0}(t_m) &{} A_{1}(t_m) &{} \cdots &{} A_{n}(t_m) \end{pmatrix}, \end{aligned}$$

and $$b_1$$ = $$[f(t_0),f(t_1),\ldots ,f(t_m)]^{T}$$.

Case:1 Consider Eq. (17) with the initial conditions. Then the following linear system is obtained:

$$\begin{aligned} \underbrace{\begin{pmatrix} A_{0}(t_0) &{} A_{1}(t_0) &{} \cdots &{} A_{n}(t_0)\\ A_{0}(t_1) &{} A_{1}(t_1) &{} \cdots &{} A_{n}(t_1)\\ \vdots &{} \vdots &{} \ddots &{} \vdots \\ A_{0}(t_m) &{} A_{1}(t_m) &{} \cdots &{} A_{n}(t_m)\\ P_{0}(a) &{} P_{1}(a) &{} \cdots &{} P_{n}(a)\\ P'_{0}(a) &{} P'_{1}(a) &{} \cdots &{} P'_{n}(a) \end{pmatrix} }_{A} \underbrace{\begin{pmatrix} w_0 \\ w_1 \\ \vdots \\ w_n \\ \end{pmatrix} }_{{\textbf {w}}} \approx \underbrace{\begin{pmatrix} f_0 \\ f_1 \\ \vdots \\ f_m \\ z_0\\ z_1\\ \end{pmatrix} }_{{\textbf {b}}} \end{aligned}$$

Case:2 Consider Eq. (17) with the boundary conditions. Then the following linear system for NDDE is obtained:

$$\begin{aligned} \underbrace{\begin{pmatrix} A_{0}(t_0) &{} A_{1}(t_0) &{} \cdots &{} A_{n}(t_0)\\ A_{0}(t_1) &{} A_{1}(t_1) &{} \cdots &{} A_{n}(t_1)\\ \vdots &{} \vdots &{} \ddots &{} \vdots \\ A_{0}(t_m) &{} A_{1}(t_m) &{} \cdots &{} A_{n}(t_m)\\ P_{0}(a) &{} P_{1}(a) &{} \cdots &{} P_{n}(a)\\ P_{0}(b) &{} P_{1}(b) &{} \cdots &{} P_{n}(b) \end{pmatrix} }_{A} \underbrace{\begin{pmatrix} w_0 \\ w_1 \\ \vdots \\ w_n \\ \end{pmatrix} }_{{\textbf {w}}} \approx \underbrace{\begin{pmatrix} f_0 \\ f_1 \\ \vdots \\ f_m \\ z_2\\ z_3\\ \end{pmatrix} }_{{\textbf {b}}} \end{aligned}$$

To calculate the weight vector ${\textbf {w}}$ of the network, we use the extreme learning algorithm, that is:

27 $$\begin{aligned} {\textbf {w}}=A^{\dagger}b, \end{aligned}$$

where $A^{\dagger}=(A^{T}A)^{-1}A^{T}$ is the least square solution of Eq. (27).

*Note:* Similar methodology can be used for the higher order neutral delay differential equation and the system of higher order neutral delay differential equations.
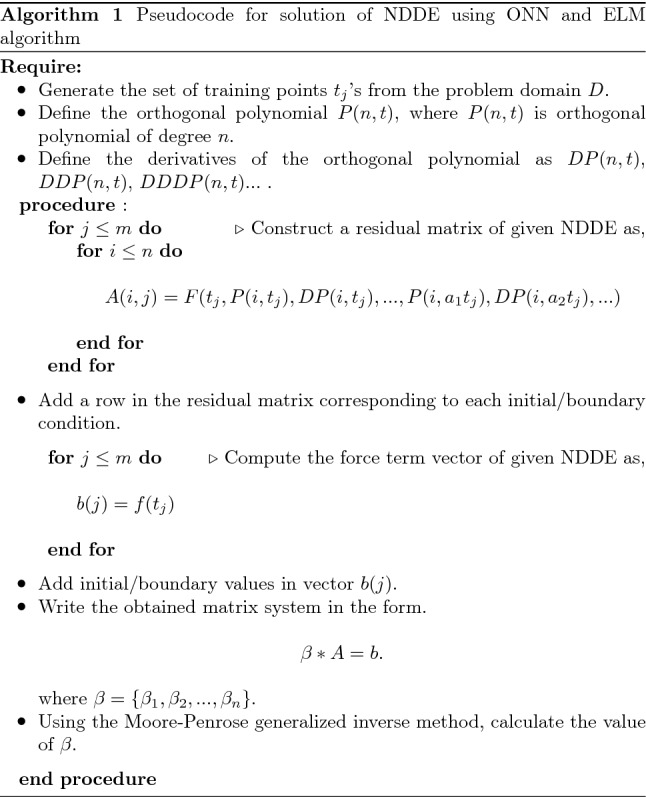

- **Steps of solving NDDEs using an ONN-ELM algorithm:**

1. Discretize the domain as $a=t_0<t_1<t_2<...<t_m=b$.
2. Construct the approximate solution by using the orthogonal polynomial as an activation function that is,
  $$\begin{aligned} N(t,\textbf{w})= \sum _{i=0}^{n} w_{i} P_{i}(a_{i}t), \end{aligned}$$
  where $a_i's$ are the randomly generated fixed weights.
3. At the discrete points, substitute the approximate solution and its derivatives into the differential equation and its boundary conditions and obtain the system of equations $\textbf{A}\textbf{w}=\textbf{b}$.
4. Solve the system of equations $\textbf{A}\textbf{w}=\textbf{b}$ by ELM algorithm and obtain the network weights $w_i's$.
5. Substitute the value of $w_i's$ and get an approximate solution of DDE.

## Numerical illustrations

This section considers the higher order delay and neutral delay differential equations with multiple delays and variable coefficients. We also consider the system of delay and neutral delay differential equations. In all the test examples, we use the special orthogonal polynomials based neural network like Legendre neural network, Laguerre neural network, Chebyshev neural network, and Hermite neural network. Further, to show the reliability and powerfulness of the presented method; we compare the approximate solutions with the exact solution. All computations are carried out using Python 3.9.7 on Intel(R) Core(TM) i5-8250U CPU @ 1.60GHz 1.80 GHz and the Window 10 operating system. We calculate the relative error which is defined as follows.

$$\begin{aligned} Relative \; error = \left\| \frac{exact \; solution - numerical \; solution}{exact \; solution} \right\| \end{aligned}$$

### *Example 6.1*

^22^ Consider the second-order boundary valued proportional delay differential equation with variable coefficients

$$\begin{aligned} \begin{aligned} z''(t)&=0.5z(t)+e^{0.5t}z\left( \frac{t}{2}\right) -2e^{-t},\\ z(0)&=0, \,\,\, z(1)=e^{-1}. \end{aligned} \end{aligned}$$

The exact solution of the given equation is $te^{-t}$.

**Figure 2:**
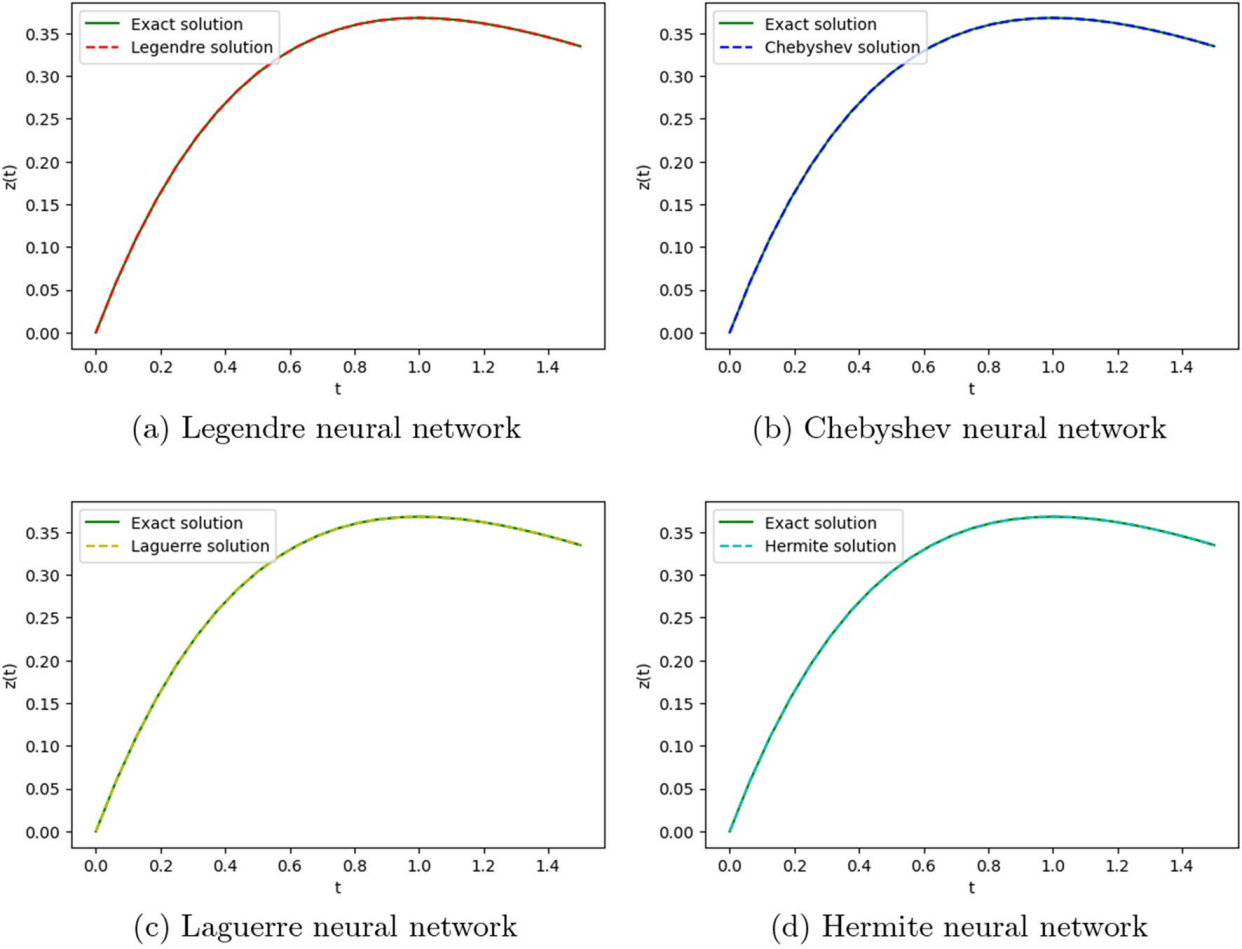
Comparison of the exact solution with the obtained approximate solutions of Example 6.1.

**Figure 3:**
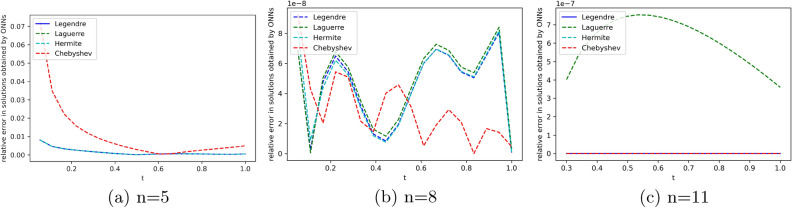
Error graph for different orthogonal neural networks with different numbers of neurons for Example 6.1.

We employ four ONNs to obtain the approximate solution of the given second-order DDE with variable coefficients. We choose ten uniformly distributed points in [0, 1]. The relative errors for all ONNs are shown in Fig. 3. Obtained relative errors for different orthogonal neural networks are reported in Table 1, and we compare the approximate solutions with the exact solution in Fig. 2.

Table 1 and Fig. 3 clearly show that the Chebyshev polynomial-based ONN performs best with the maximum relative error $5.61\times 10^{-8}$. Table 2 shows the comparison of the maximum relative error for Example 6.1 using the Legendre, Laguerre, Hermite, and Chebyshev neural networks with various numbers of neurons (n = 5, 8, and 11) and their respective computational time. Additionally, Table 2 shows that all four neural networks satisfy Theorem 4, and for $n=5$, all four orthogonal neural networks show similar accuracy. However, Chebyshev neural network performs better with $n=8,11$.

**Table 1:** The relative error for Example 6.1 with different orthogonal neural networks.

t	Legendre neural network	Hermite neural network	Laguerre neural network	Chebyshev neural network
0.1	1.56e−08	2.26e−08	1.39e−08	5.61e−08
0.2	6.20e−08	5.89e−08	6.49e−08	4.54e−08
0.3	4.57e−08	4.39e−08	4.89e−08	4.12e−08
0.4	1.07e−08	9.71e−09	1.40e−08	2.09e−08
0.5	1.94e−08	1.88e−08	2.26e−08	4.57e−08
0.6	5.66e−08	5.64e−08	5.98e−08	1.06e−08
0.7	6.84e−08	6.86e−08	7.17e−08	2.73e−08
0.8	5.09e−08	5.14e−08	5.42e−08	1.32e−08
0.9	6.99e−08	7.08e−08	7.34e−08	1.79e−08
1	7.08e−10	4.55e−10	2.93e−09	4.63e−09

**Table 2:** Comparision of maximum relative error for Example 6.1 with different numbers of neurons.

n	Legendre		Laguerre		Hermite		Chebyshev	
	Time(s)	Error	Time(s)	Error	Time(s)	Error	Time(s)	Error
5	0.004	**0.0049**	0.009	**0.0049**	0.002	**0.0049**	0.008	**0.0049**
8	0.012	**7.07e−08**	0.011	6.97e−08	0.003	**7.07e−08**	0.043	**7.07e−08**
11	0.019	1.82e−11	0.011	7.74e−06	0.003	6.81e−10	0.043	**3.93e−12**

Significant values are in bold.

### *Example 6.2*

^2^ Consider the second-order neutral delay differential equation with multiple delays

$$\begin{aligned} \begin{aligned} z''(t)&= \frac{3}{4}z(t)+z\left( \frac{t}{2}\right) +z'\left( \frac{t}{2}\right) +0.5z''\left( \frac{t}{2}\right) +f(t),\\ z(0)&=0,\, z'(0)=0, \end{aligned} \end{aligned}$$

where $$f(t)=-t^{2}-t+1$$, $$t\in (0,1)$$.

The exact solution of the given equation is $z(t)=t^{2}$.

**Figure 4:**
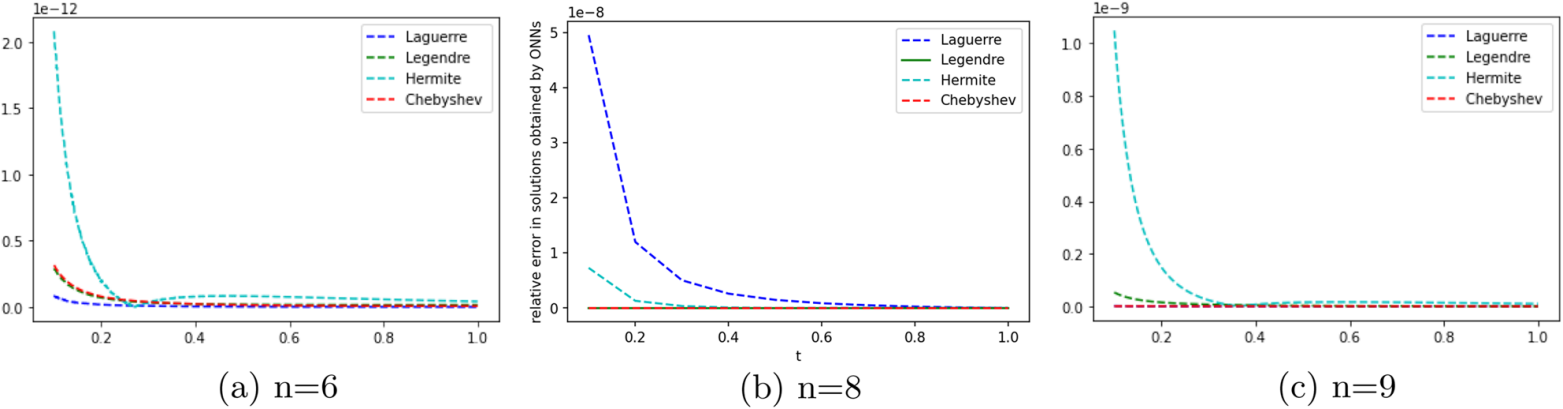
Error graph for different orthogonal neural networks with different numbers of neurons for Example 6.2.

**Figure 5:**
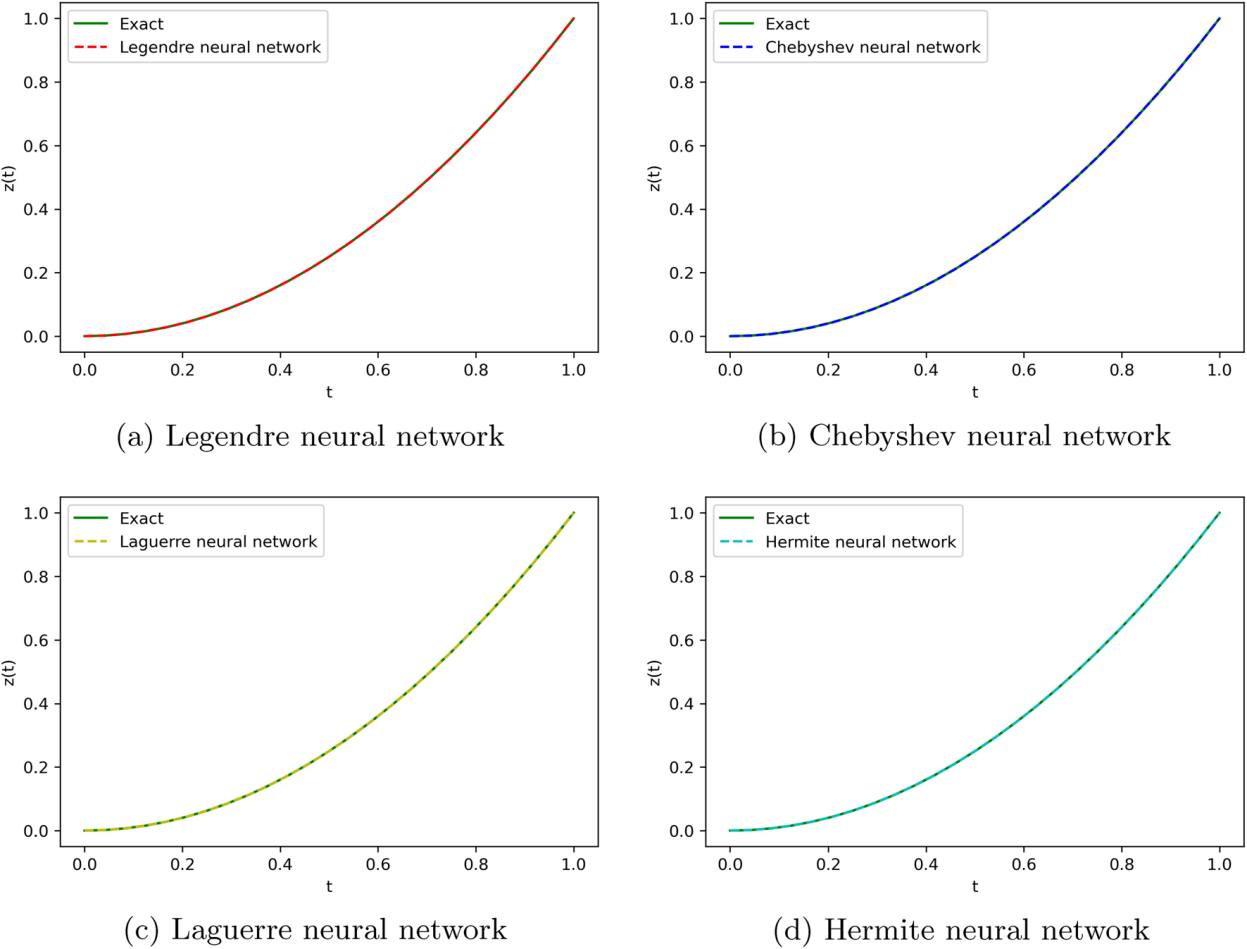
Comparison of the exact solution with the obtained approximate solutions of Example 6.2.

This equation is solved using four ONNs architecture with ten uniformly distributed training points and with 6,8, and 9 neurons in the hidden layer. Relative errors for the different ONNs with 6,8, and 9 neurons as activation functions are reported in Table 3. Figure 4 shows an error graph of different orthogonal neural networks, and a comparison of approximate solutions with the exact solution is shown in Fig. 5.

From Table 4 and Fig. 4 we conclude that for the given second-order neutral delay differential equation, Chebyshev polynomial-based ONN performs best with the maximum relative error $7.19\times 10^{-14}$. Additionally, Table 3 shows that all four neural networks satisfy Theorem 4.

**Table 3:** Comparision of maximum relative error for Example 6.2 with different numbers of neurons.

n	Legendre		Laguerre		Hermite		Chebyshev	
	Time(s)	Error	Time(s)	Error	Time(s)	Error	Time(s)	Error
6	0.007	**8.25e−14**	0.004	2.06e−12	0.002	2.87e−13	0.001	3.17e−14
8	0.009	4.94e−08	0.004	7.29e−09	0.004	7.73e−13	0.002	**7.19e−14**
9	0.019	2.22e−14	0.069	1.57e−08	0.017	6.13e−13	0.022	**6.77e−15**

Significant values are in bold.

**Table 4:** The relative error for Example 6.2 with different orthogonal neural networks.

t	Legendre neural network	Hermite neural network	Laguerre neural network	Chebyshev neural network
0.1	4.94e−08	7.73e−13	7.29e−09	7.19e−14
0.2	1.19e−08	1.90e−13	1.32e−09	1.00e−14
0.3	5.05e−09	7.61e−14	3.83e−10	9.25e−15
0.5	1.49e−09	1.26e−14	8.31e−12	5.55e−15
0.6	8.88e−10	3.08e−16	3.37e−11	6.32e−15
0.7	5.20e−10	7.81e−15	5.12e−11	7.13e−15
0.8	2.81e−10	1.37e−14	5.75e−11	8.15e−15
0.9	1.17e−10	1.80e−14	5.85e−11	9.45e−15
1	2.66e−15	2.17e−14	5.68e−11	1.11e−14

### *Example 6.3*

^2^ Consider the second-order neutral delay differential equation with variable coefficients

$$\begin{aligned} \begin{aligned} z''(t)&=z'\left( \frac{t}{2}\right) -\frac{t}{2}z''\left( \frac{t}{2}\right) +2, \, t \in (0,1)\\ z(0)&=1,\, z'(0)=0. \end{aligned} \end{aligned}$$

The exact solution of the given equation is $z(t)=t^2+1$.

**Figure 6:**
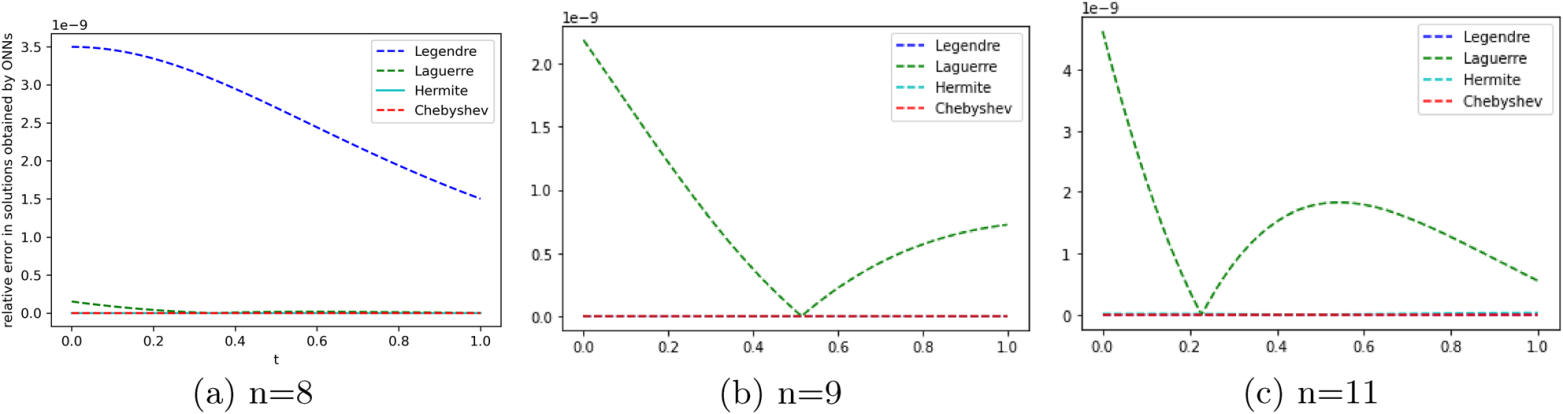
Error graph for different orthogonal neural networks with different numbers of neurons for Example 6.3.

**Figure 7:**
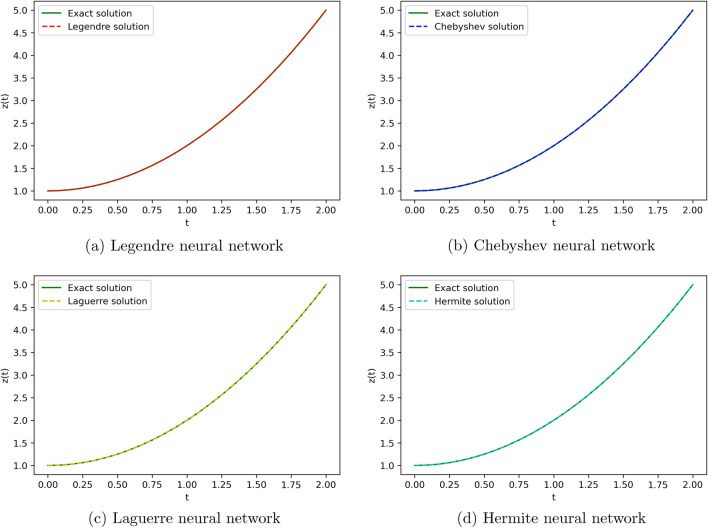
Comparison of the exact solution with the obtained approximate solutions of Example 6.3.

To obtain the approximate solution of the given equation, we use four ONNs with ten uniformly distributed training points in [0,1] and with 8,9, and 11 neurons as activation functions in the hidden layer. Relative errors for the different ONNs and with different numbers of neurons are reported in Table 6. The exact and approximate solutions are compared in Fig. 7. Figures 6, 7 shows the absolute relative error of four special ONNs.

From Table 5 and Fig. 6, we conclude that for the given second-order neutral delay differential equation, Chebyshev polynomial-based ONN provides the best accurate solution with the maximum relative error $2.29\times 10^{-15}$. Additionally, Table 6 shows that all four neural networks satisfy Theorem 4.

**Table 5:** The relative error for Example−6.3 with different orthogonal neural networks.

t	Legendre neural network	Hermite neural network	Laguerre neural network	Chebyshev neural network
0.0	3.50e−09	1.17e−13	1.52e−10	7.77e−16
0.1	3.46e−09	1.14e−13	8.90e−11	0.0
0.2	3.34e−09	1.01e−13	4.18e−11	1.06e−15
0.3	3.16e−09	8.06e−14	1.01e−11	1.83e−15
0.4	2.94e−09	5.28e−14	8.50e−12	2.29e−15
0.5	2.70e−09	1.98e−14	1.72e−11	2.13e−15
0.6	2.44e−09	1.58e−14	1.90e−11	1.63e−15
0.7	2.18e−09	5.27e−14	1.66e−11	1.04e−15
0.8	1.93e−09	8.93e−14	1.21e−11	2.70e−16
0.9	1.70e−09	1.24e−13	7.09e−12	4.90e−16
1.0	1.50e−09	1.52e−13	2.35e−12	8.88e−16

**Table 6:** Comparision of maximum relative error for Example 6.3 with different numbers of neurons.

n	Legendre		Laguerre		Hermite		Chebyshev	
	Time(s)	Error	Time(s)	Error	Time(s)	Error	Time(s)	Error
8	0.007	3.50e−09	0.004	1.52e−10	0.002	1.52e−13	0.001	**2.29e−15**
9	0.009	3.07e−13	0.004	8.80e−11	0.004	1.69e−05	0.002	**2.29e−15**
11	0.019	3.07e−13	0.069	3.16e−11	0.017	1.80e−05	0.022	**1.32e−15**

Significant values are in bold.

### *Example 6.4*

^31^ Consider the third-order pantograph equation

$$\begin{aligned} \begin{aligned} z'''(t)&=tz''(2t)-z'(t)-z\left( \frac{t}{2}\right) +tcos(2t)+cos\left( \frac{t}{2}\right) , \, t \in (0,1)\\ z(0)&=1,\, z'(0)=0,\, z''(0)=-1. \end{aligned} \end{aligned}$$

The exact solution of the given equation is $z(t)=cos(t)$.

**Figure 8:**
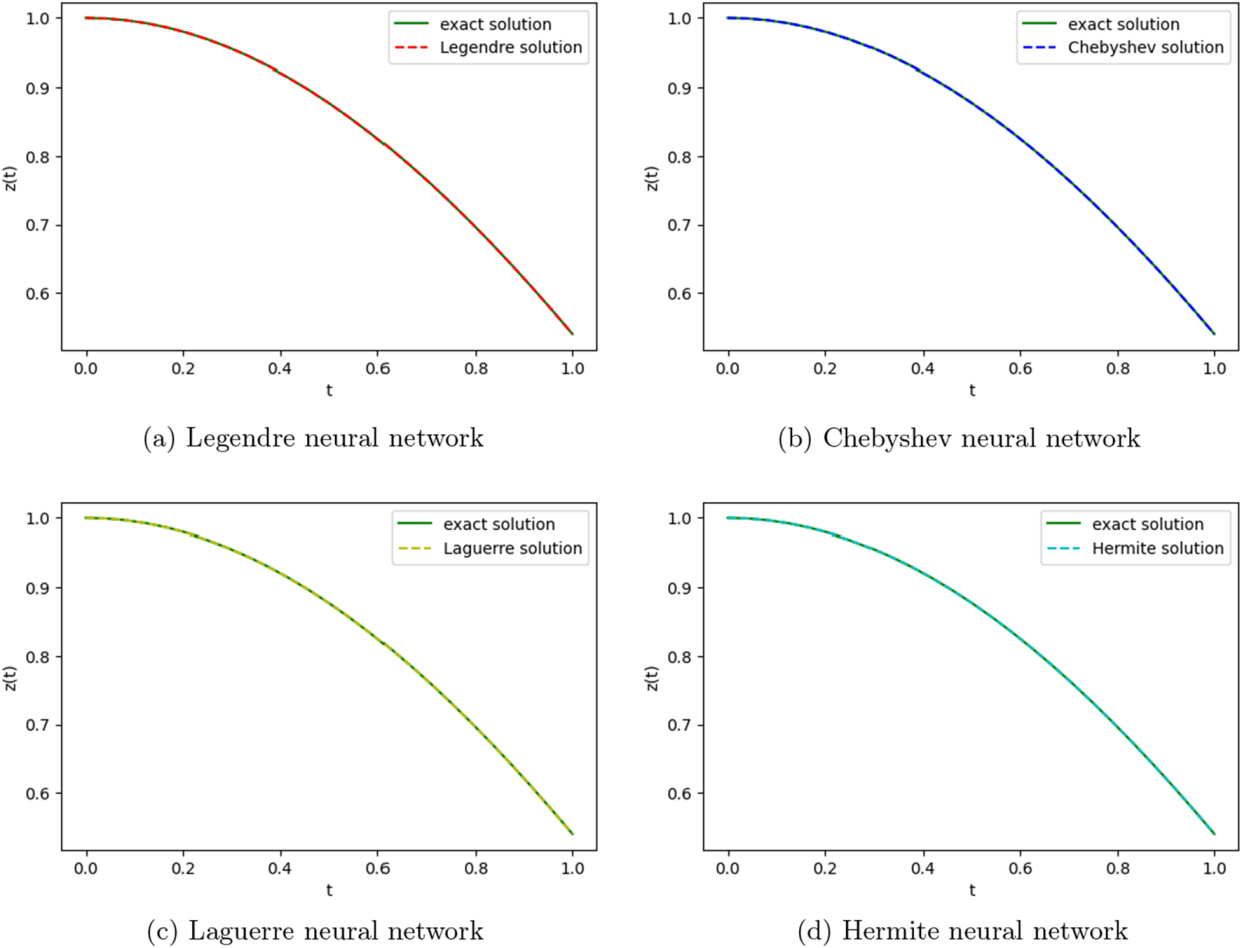
Comparison of the exact solution with the obtained approximate solutions of Example 6.4.

**Figure 9:**
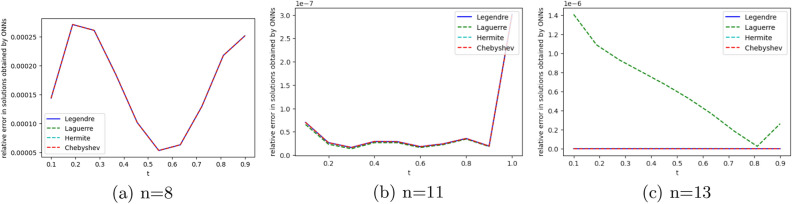
Error graph for different orthogonal neural networks with different numbers of neurons for Example 6.4.

To obtain the approximate solution of the given equation, we use four ONNs with ten uniformly distributed training points in [0,1] and with 8,11,13 neurons as activation functions in the hidden layer. Relative errors for the different ONNs with different numbers of neurons as activation functions are reported in Table 7. The exact and approximate solutions are compared in Fig. 8. Figure 9 shows the maximum relative error of four special ONNs with different numbers of neurons.

From Table 8 and Fig. 9, we conclude that for the given third-order neutral delay differential equation, Chebyshev polynomial-based ONN provides the best accurate solution with the maximum relative error $3.77\times 10^{-10}$. Additionally, Table 7 shows that all four orthogonal neural networks satisfy Theorem 4.

**Table 7:** Comparision of maximum relative error for Example 6.4 with different numbers of neurons.

n	Legendre		Laguerre		Hermite		Chebyshev	
	Time(s)	Error	Time(s)	Error	Time(s)	Error	Time(s)	Error
8	0.007	**1.11e−05**	0.004	**1.11e−05**	0.002	**1.11e−05**	0.001	**1.11e−05**
11	0.019	**3.86e−08**	0.004	6.56e−08	0.004	6.06e−08	0.004	**3.86e−08**
13	0.019	1.12e−09	0.069	3.11e−09	0.017	1.20e−06	0.022	**3.77e−10**

Significant values are in bold.

**Table 8:** The relative error for Example 6.4 with different orthogonal neural networks.

t	Legendre neural network	Hermite neural network	Laguerre neural network	Chebyshev neural network
0	3.79e−10	7.39e−07	5.91e−10	1.00e−11
0.1	3.82e−10	5.75e−07	6.05e−10	6.11e−12
0.2	3.89e−10	3.94e−07	6.33e−10	5.81e−12
0.3	4.02e−10	2.05e−07	6.75e−10	2.65e−11
0.4	4.21e−10	1.32e−08	7.33e−10	5.73e−11
0.5	4.49e−10	1.79e−07	8.11e−10	9.98e−11
0.6	4.88e−10	3.71e−07	9.22e−10	1.54e−10
0.7	5.49e−10	5.65e−07	1.10e−09	2.20e−10
0.8	6.49e−10	7.63e−07	1.41e−09	2.92e−10
0.9	8.21e−10	9.72e−07	2.00e−09	3.54e−10
1	1.12e−09	1.20e−06	3.11e−09	3.77e−10

## Comparative analysis

This section describes a comparative study of the proposed approach to the 1st-order pantograph equation and system of pantograph equations with other neural network approaches.

### *Example 7.1*

^25^ Consider the pantograph equation with variable coefficients and multiple delays

$$\begin{aligned} \begin{aligned} z'(t)&=0.5z(t)+0.5e^{0.5t}z\left( \frac{t}{2}\right) +\frac{3}{8}tz\left( \frac{t}{3}\right) +g(t),\\ z(0)&=0, \end{aligned} \end{aligned}$$

where, $$g(t)=\frac{1}{8}e^{-t}(12sin(t)+4e^{t}sin(\frac{t}{2})-8cos(t)+3te^{\frac{2t}{3}}sin(\frac{t}{3}))$$.

The exact solution of the given equation is $z(t)=sin(t)e^{-t}$.

**Figure 10:**
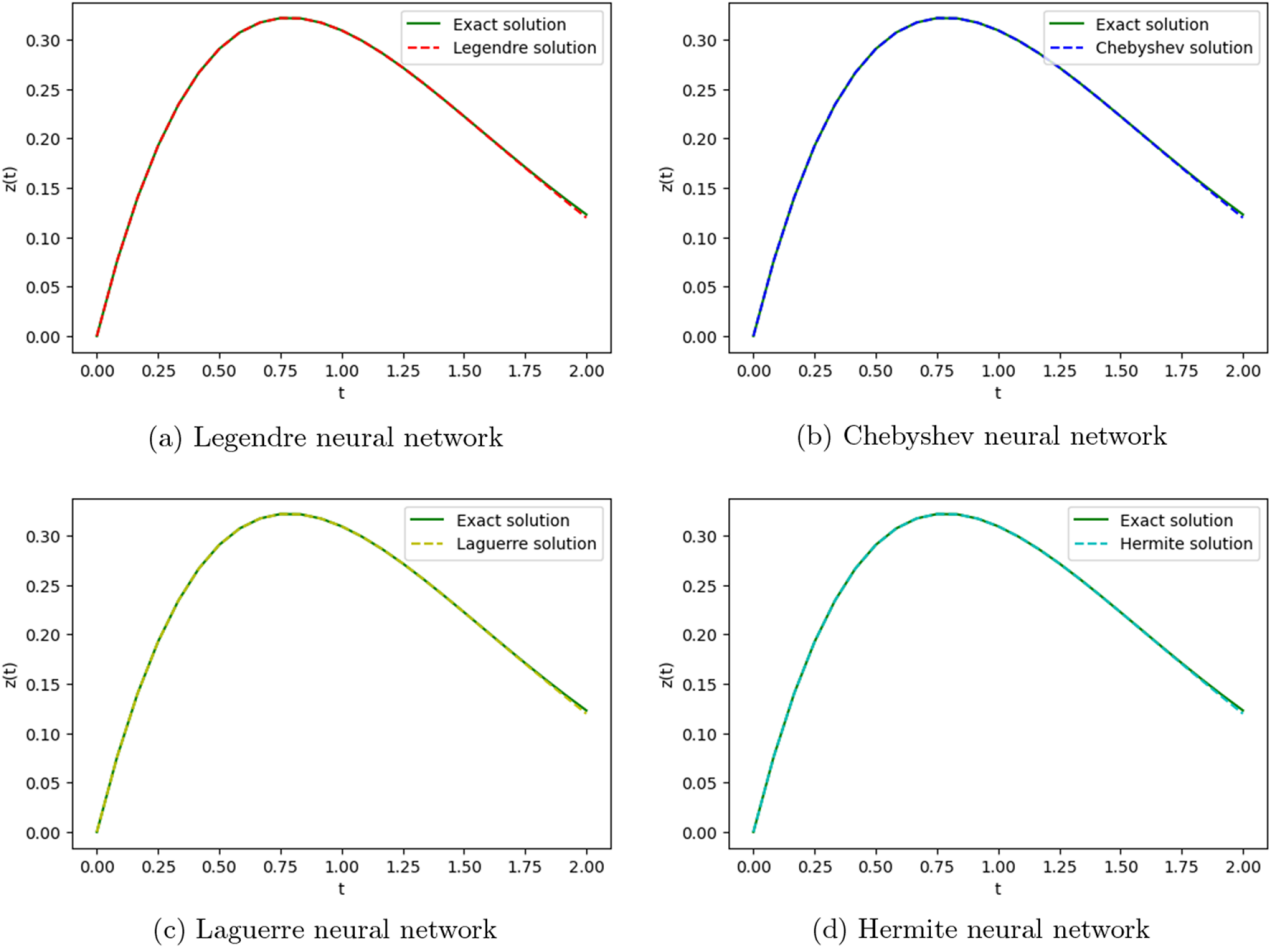
Comparison of the exact solution with the obtained approximate solutions of Example 7.1.

**Figure 11:**
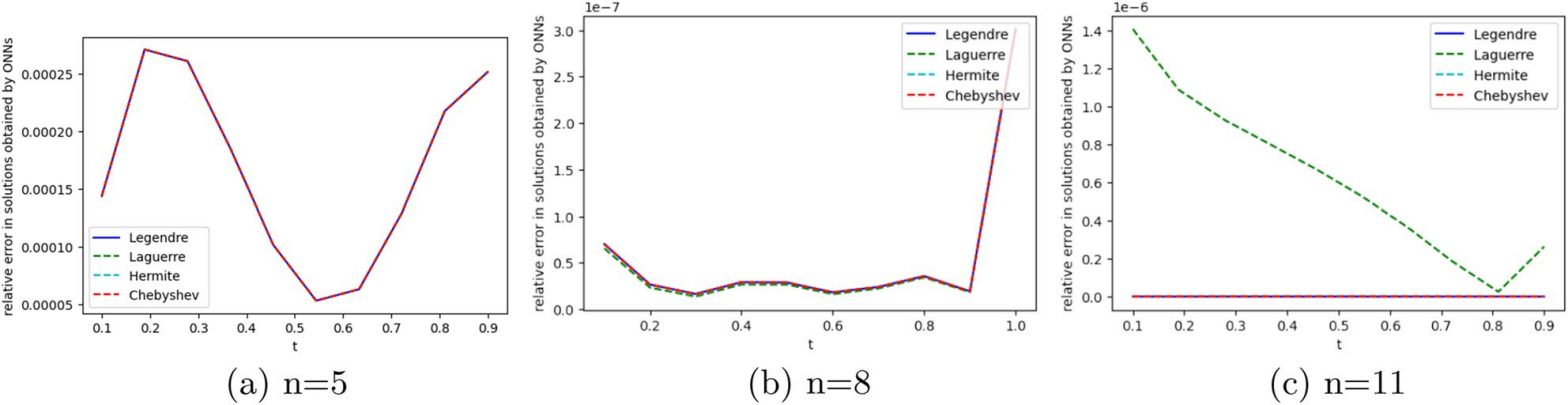
Error graph for different orthogonal neural networks with different numbers of neurons for Example 7.1.

We employ four ONNs to obtain the approximate solution of a given pantograph equation with multiple delays. We choose eight uniformly distributed points in [0, 1] with 5,8 and 11 neurons in the hidden layer. The relative errors with all four ONNs with different numbers of neurons are shown in Fig. 11. Obtained relative errors for the different orthogonal neural networks are reported in Table 9, and we compare the approximate solutions with the exact solution in Fig. 10.

Table 9 and Fig. 11 clearly show that the Chebyshev polynomial-based ONN performs best with the maximum relative error $3.40\times 10^{-11}$.

The maximum relative error of a simple feed-forward neural network(FNN) method in^25^ is $4.05\times 10^{-10}$ and the maximum relative error of the proposed FLNN-based ONN method is $3.40\times 10^{-11}$. This comparison shows that the ONN method can obtain a better accuracy solution than simple FNN. Additionally, Table 9 shows that all four orthogonal neural networks satisfy Theorem 4.

**Table 9:** Comparision of maximum relative error for Example 7.1 with different numbers of neurons.

n	Legendre		Laguerre		Hermite		Chebyshev	
	Time(s)	Error	Time(s)	Error	Time(s)	Error	Time(s)	Error
5	0.007	**0.0014**	0.004	**0.0014**	0.002	**0.0014**	0.001	**0.0014**
8	0.019	**7.02e−08**	0.004	6.56e−08	0.004	**7.02e−08**	0.004	**7.02e−08**
11	0.019	4.75e−11	0.069	1.06e−06	0.017	2.63e−09	0.022	**3.40e−11**

Significant values are in bold.

### *Example 7.2*

^25^ Consider the system of pantograph equation

$$\begin{aligned} \begin{aligned} z_{1}'(t)&=z_{1}(t)-z_{2}(t)+z_{1}\left( \frac{t}{2}\right) -e^{0.5t}+e^{t}, \\ z_{2}'(t)&=-z_{1}(t)-z_{2}(t)-z_{2}\left( \frac{t}{2}\right) +e^{-0.5t}+e^{t},\\ z_{1}(0)&=1,\, z_{2}(0)=1. \end{aligned} \end{aligned}$$

The exact solutions of the given system of pantograph equation is $z_1(t)=e^{t}$ and $z_2(t)=e^{-t}$.

**Figure 12:**
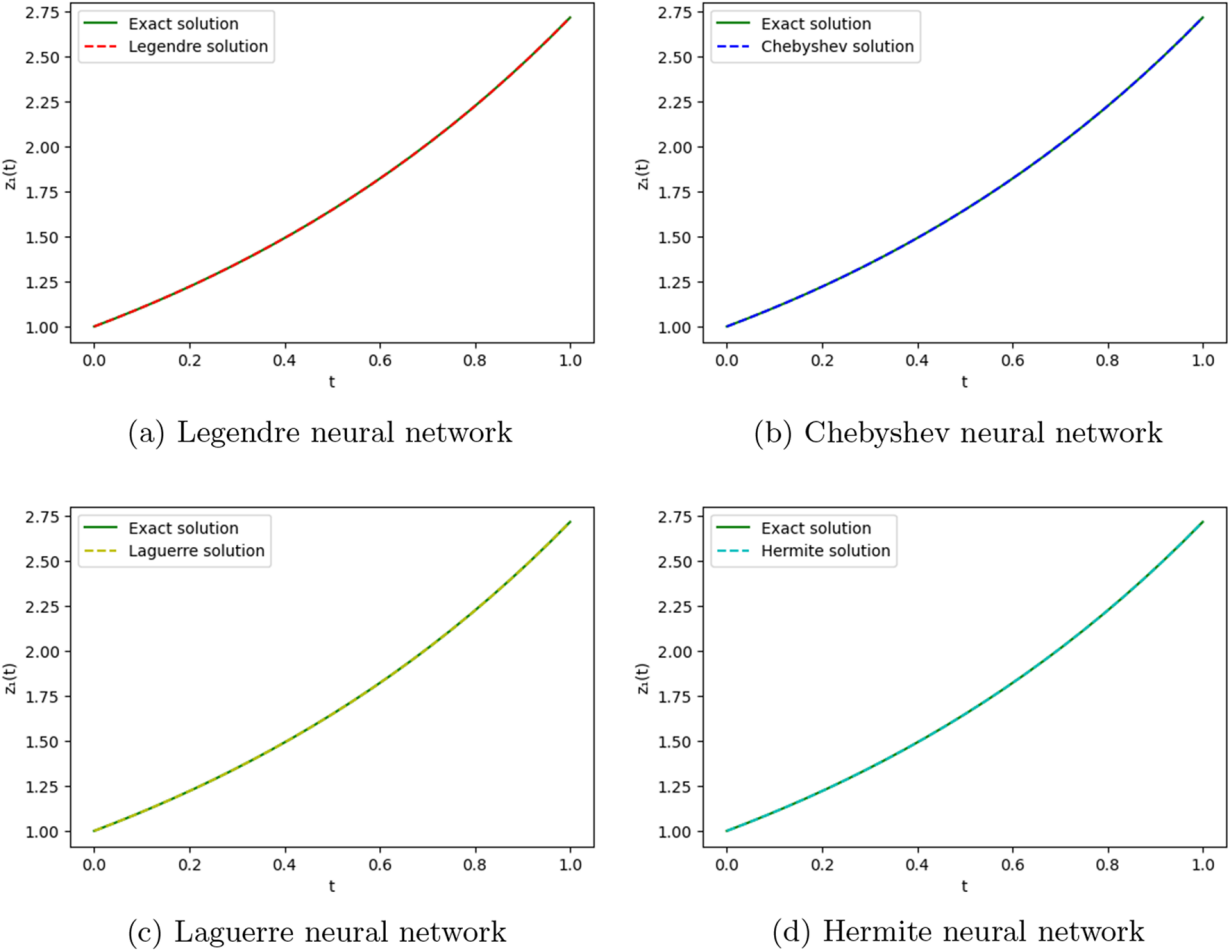
Comparison of the exact solution $z_1(t)$ with the obtained approximate solutions of Example 7.2.

**Figure 13:**
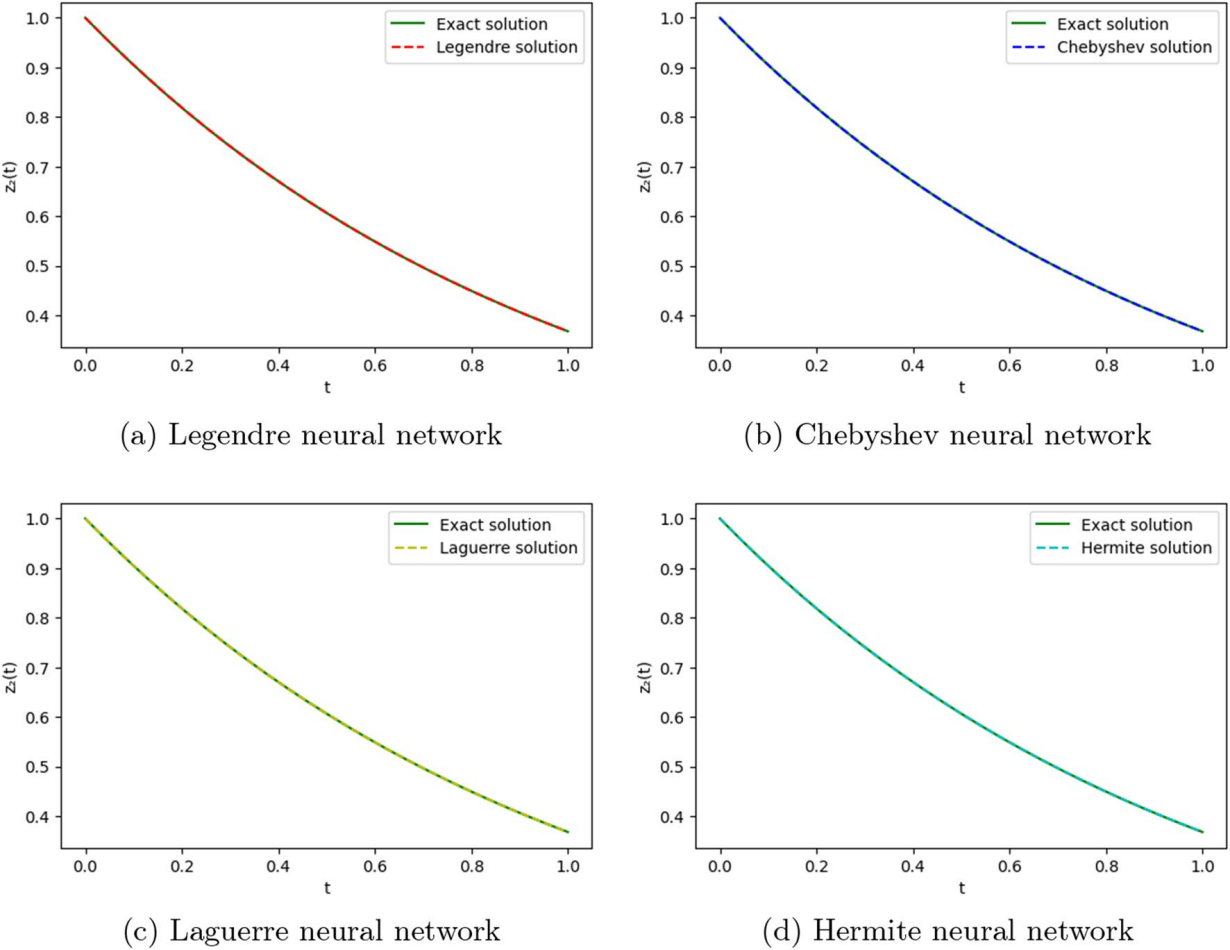
Comparison of the exact solution $z_2(t)$ with the obtained approximate solutions of Example 7.2.

**Figure 14:**
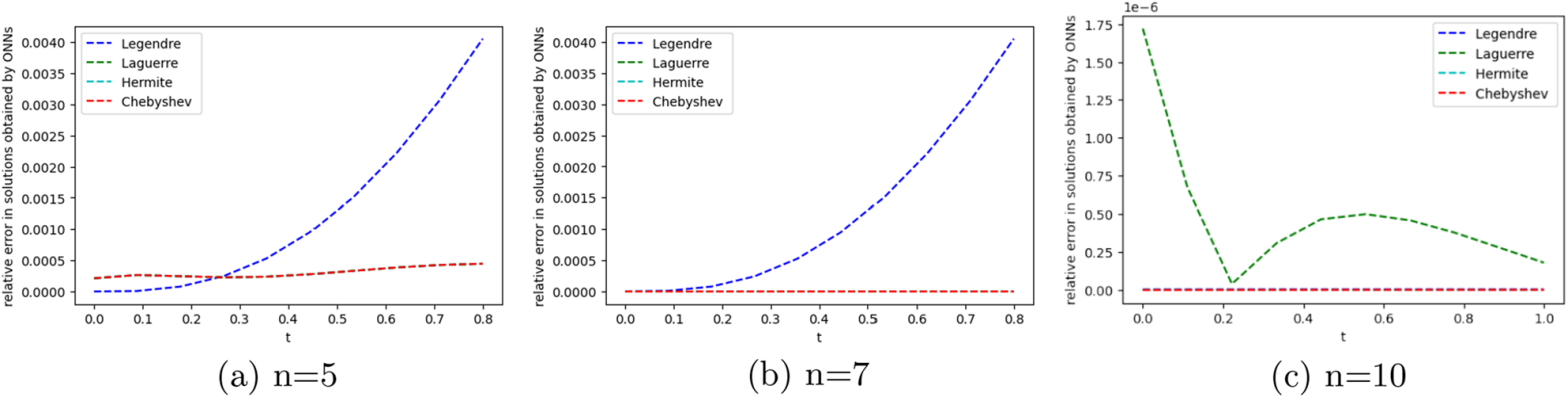
Error graph of $z_1(t)$ for different orthogonal neural networks with different numbers of neurons for Example 7.2.

**Figure 15:**
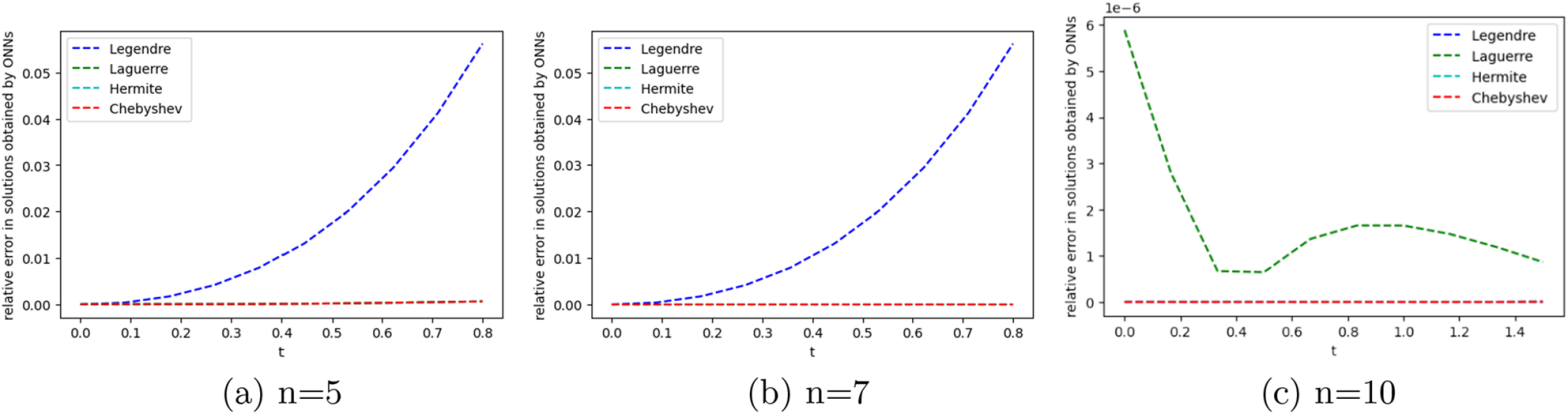
Error graph of $z_2(t)$ for different orthogonal neural networks with different numbers of neurons for Example 7.2.

To obtain the approximate solutions of the given system of DDEs, we use four ONNs with twelve uniformly distributed training points in [0,1] and with 5,7, and 10 neurons in an orthogonal functional expansion block as activation functions. Relative errors for the different ONNs with 5,7, and 10 neurons as activation functions are reported in Tables 10 and 11. Comparison between the exact solution and approximate solutions are presented in Figs. 14 and 15. Figures 12, 13, 14 and 15 show the absolute relative error between four special ONNs and exact solutions.

From Tables 10 and 11, we conclude that for the given system of delay differential equation, Chebyshev polynomial-based ONN provides the best accurate solution for $z_1(t)$ and $z_2(t)$ with the maximum relative errors $1.60\times 10^{-9}$ and $5.11\times 10^{-11}$, respectively.

The maximum relative error of a simple feed-forward neural network(FNN) method in^25^ for $z_1(t)$ and $z_2(t)$ with twelve training points are $1.93\times 10^{-9}$ and $2.42\times 10^{-9}$ respectively and the maximum relative error of the proposed FLNN-based ONN method for $z_1(t)$ and $z_2(t)$ with twelve training points are $1.60\times 10^{-9}$ and $5.11\times 10^{-10}$ respectively. This comparison shows that the ONN method can obtain a better accuracy solution than simple FNN. Additionally, Tables 10 and 11 show that all four orthogonal neural networks satisfy Theorem 4.

**Table 10:** Comparision of maximum relative error of $z_1(t)$ for Example 7.2 with different numbers of neurons.

n	Legendre		Laguerre		Hermite		Chebyshev	
	Time(s)	Error	Time(s)	Error	Time(s)	Error	Time(s)	Error
5	0.005	0.06	0.004	**0.0004**	0.002	**0.0004**	0.001	**0.0004**
7	0.019	0.067	0.004	1.72e−06	0.004	**1.93e−07**	0.004	**1.93e−07**
10	0.019	3.23e−10	0.069	1.71e−06	0.017	1.81e−09	0.022	**1.60e−10**

Significant values are in bold.

**Table 11:** Comparision of maximum relative error of $z_2(t)$ for Example 7.2 with different numbers of neurons.

n	Legendre		Laguerre		Hermite		Chebyshev	
	Time(s)	Error	Time(s)	Error	Time(s)	Error	Time(s)	Error
5	0.005	0.312	0.004	**0.0006**	0.002	**0.0006**	0.001	**0.0006**
7	0.019	0.02	0.004	1.94e−06	0.004	2.00e−07	0.004	**6.47e−09**
10	0.019	1.42e−08	0.069	2.20e−08	0.017	5.91e−06	0.022	**5.11e−10**

Significant values are in bold.

### *Example 7.3*

^25^ Consider the system of pantograph equation

$$\begin{aligned} \begin{aligned} z_{1}'(t)+z_{2}'(t)-2z_{3}'(t)&=z_{1}(0.2t)+z_{2}(t)-z_{2}(0.3t)\\ & \quad -2z_{3}(t)-z_{3}(0.3t)+z_{3}(0.5)+f_1(t), \\ z_{1}'(t)-z_{2}'(t)&=z_{1}(t)-z_{3}(t)+3z_{1}(0.5t)\\ & \quad -z_{2}(0.5t)+z_{2}(0.3t)+z_{3}(0.7t)+f_2(t),\\ z_{2}'(t)-2z_{3}'(t)&=z_{1}(t)-z_{3}(0.8t)+3z_{2}(t)\\ & \quad -z_{1}(0.2t)+z_{3}(0.8t)+f_3(t),\\ z_{1}(0)&=0,\, z_{2}(0)=1, z_{3}(0)=1, \end{aligned} \end{aligned}$$

where, $$f_{1}(t)=cos(0.3t)-sin(0.2t)-sin(t)+e^{0.3t}-e^{0.5t}$$,

$$f_{2}(t)=-cos(0.3t)+cos(0.5t)-3sin(0.5t)+cos(t)-e^{0.7t}+e^{t}$$,

$$f_{3}(t)=-cos(0.8t)+sin(0.2t)-3cos(t)-2sin(t)+e^{0.8t}-2e^{t}$$.

The exact solutions of the given system of pantograph equation are $z_1(t)=sin(t)$, $z_2(t)=cos(t)$, and $z_3(t)=e^{t}$.

**Figure 16:**
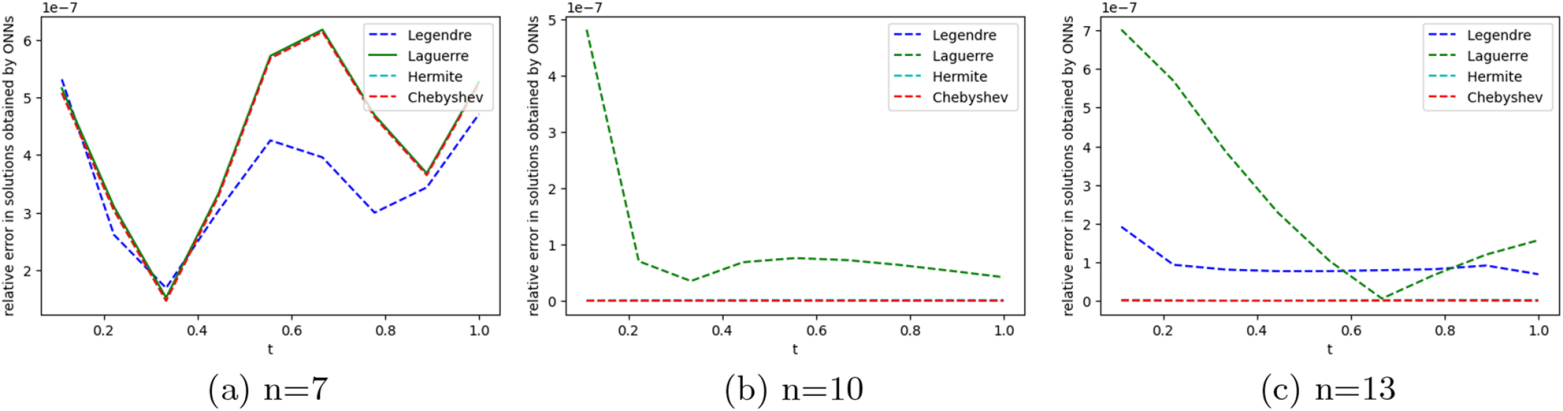
Error graph of $z_1(t)$ for different orthogonal neural networks with different numbers of neurons for Example 7.3.

**Figure 17:**
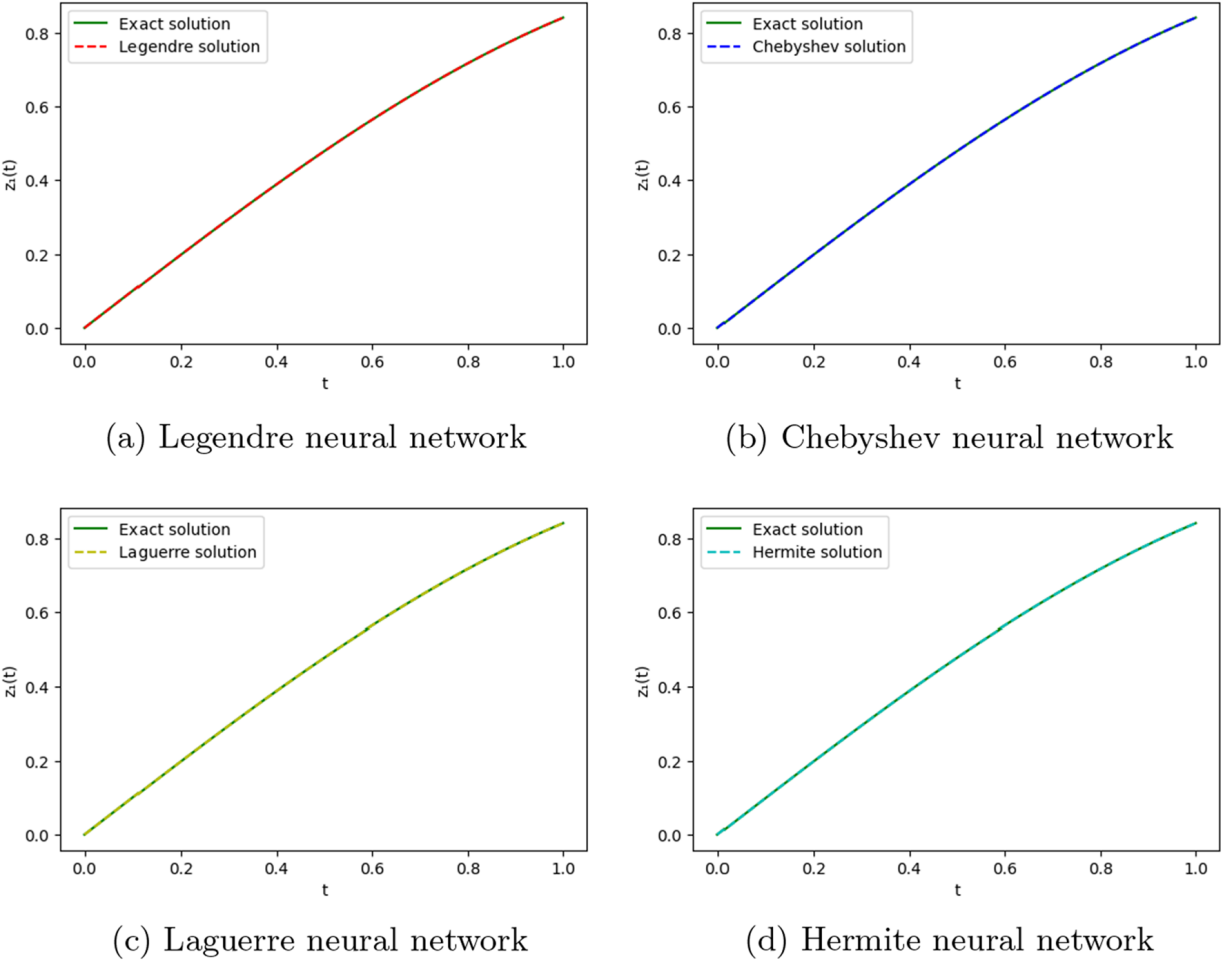
Comparison of the exact solution $z_1(t)$ with the obtained approximate solutions of Example 7.3.

**Figure 18:**
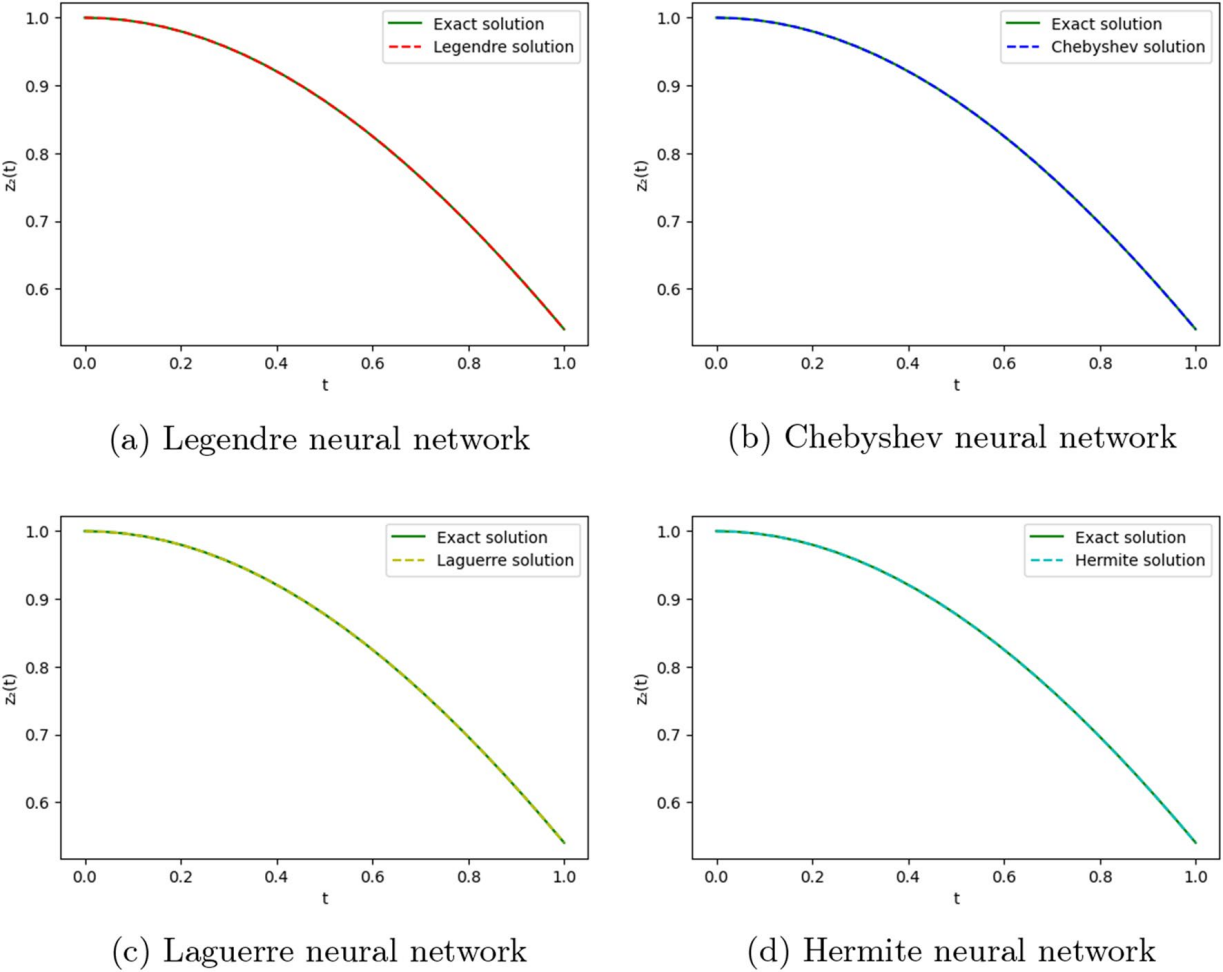
Comparison of the exact solution $z_2(t)$ with the obtained approximate solutions of Example 7.3.

**Figure 19:**
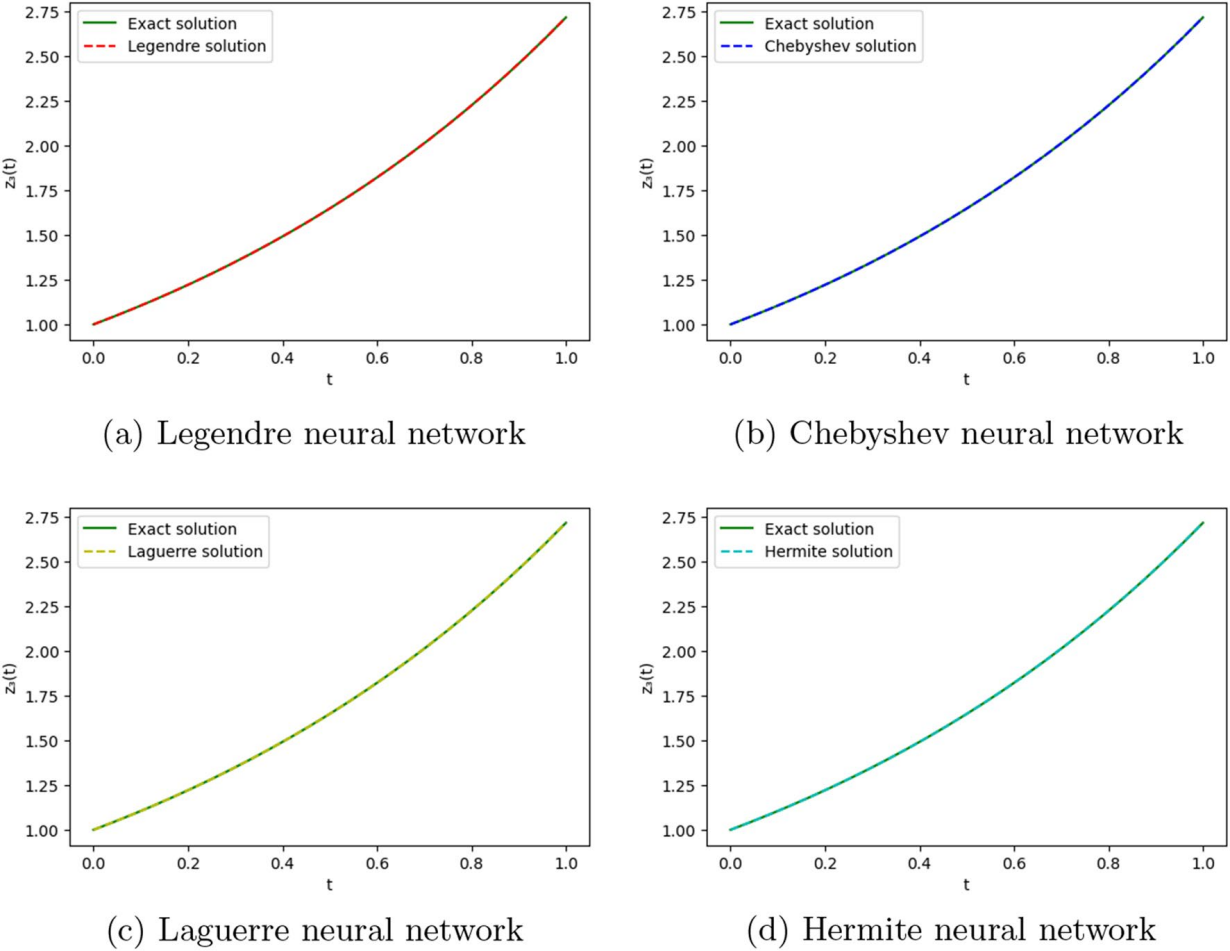
Comparison of the exact solution $z_3(t)$ with the obtained approximate solutions of Example 7.3.

**Figure 20:**
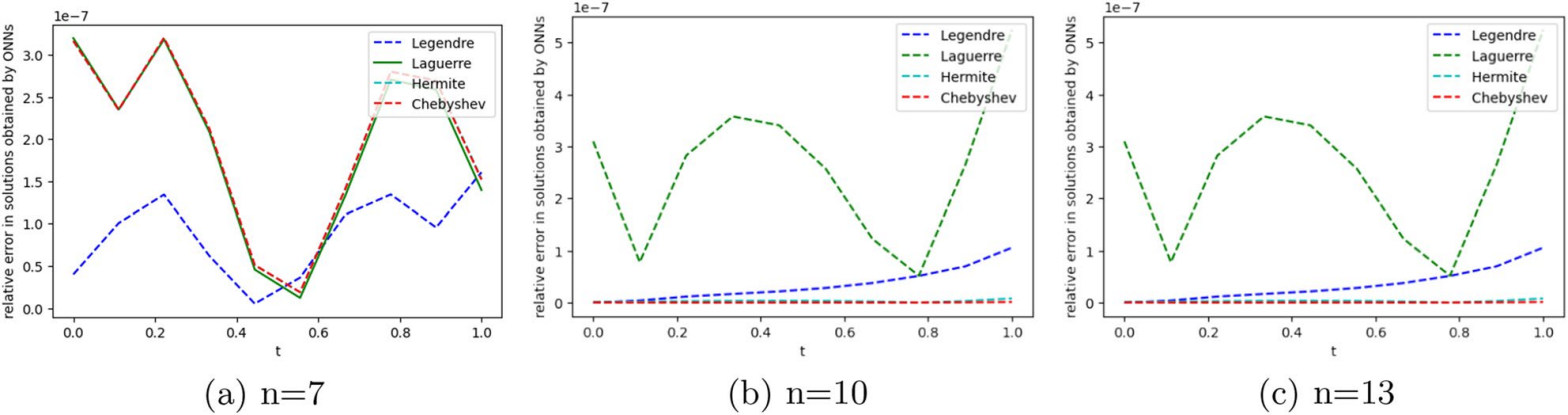
Error graph of $z_2(t)$ for different orthogonal neural networks with different numbers of neurons for Example 7.3.

**Figure 21:**
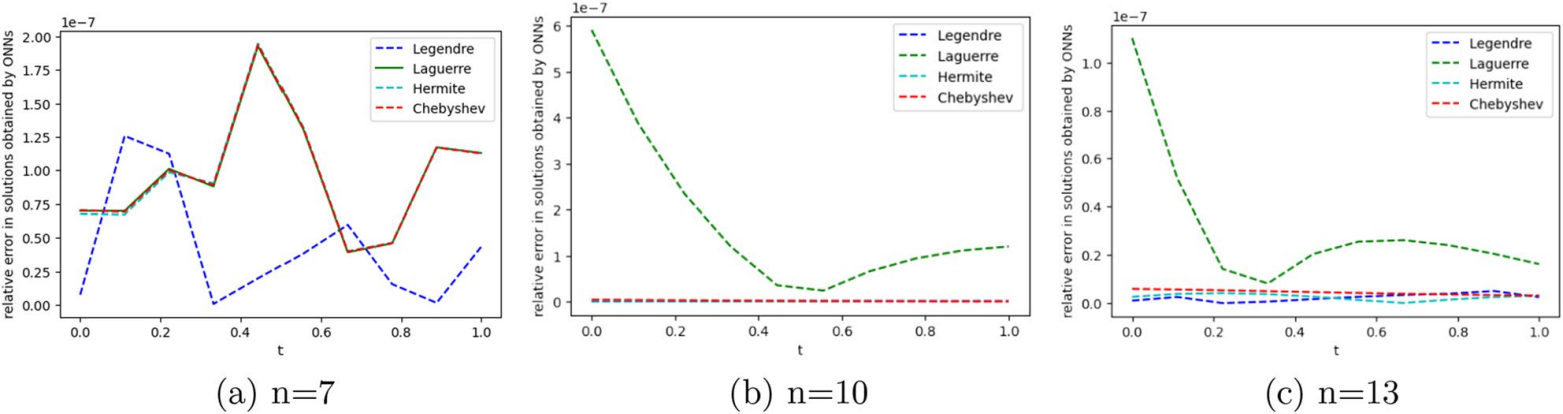
Error graph of $z_3(t)$ for different orthogonal neural networks with different numbers of neurons for Example 7.3.

To obtain the approximate solution of the given system of DDEs, we use four ONNs with ten uniformly distributed training points in [0,1] and with 7,10, and 13 neurons in an orthogonal functional expansion block as activation functions. Relative errors for the different ONNs with 7,10, and 13 neurons as activation functions are reported in Tables 12, 13, and 14. Comparison between the exact solution and approximate solutions are presented in Figs. 16, 17, 18, and 19. Figures 16, 20, and 21 show the absolute relative error between four special ONNs and exact solutions.

From Tables 12, 13 and 14, we conclude that for the given system of delay differential equation, Chebyshev polynomial-based ONN provides the best accurate solutions of $z_1(t)$,$z_2(t)$ and $z_3(t)$ with the maximum relative errors $1.98\times 10^{-10}$, $3.11\times 10^{-10}$ and $5.74\times 10^{-9}$ respectively.

The maximum relative error of a simple feed-forward neural network(FNN) method in^25^ for $z_1(t)$, $z_2(t)$ and $z_3(t)$ with ten training points are $8.78\times 10^{-8}$, $1.42\times 10^{-8}$ and $1.93\times 10^{-7}$ respectively and the maximum relative error of the proposed FLNN-based ONN method for $z_1(t)$, $z_2(t)$ and $z_3(t)$ with ten training points are $1.98\times 10^{-10}$, $3.11\times 10^{-10}$ and $5.74\times 10^{-9}$ respectively. This comparison shows that the ONN method can obtain a better accuracy solution than simple FNN. Additionally, Tables 12, 13 and 14 show that all four orthogonal neural networks satisfy Theorem 4.

**Table 12:** Comparision of maximum relative error of $z_1(t)$ for Example 7.3 with different numbers of neurons.

n	Legendre		Laguerre		Hermite		Chebyshev	
	Time(s)	Error	Time(s)	Error	Time(s)	Error	Time(s)	Error
7	0.007	**5.41e−07**	0.004	6.20e−07	0.002	6.17e−07	0.001	6.16e−07
10	0.019	9.87e−08	0.004	6.97e−07	0.004	1.45e−09	0.004	**1.53e−10**
13	0.019	9.11e−11	0.069	5.79e−07	0.017	1.23e−09	0.022	**1.98e−11**

Significant values are in bold.

**Table 13:** Comparision of maximum relative error of $z_2(t)$ for Example 7.3 with different numbers of neurons.

n	Legendre		Laguerre		Hermite		Chebyshev	
	Time(s)	Error	Time(s)	Error	Time(s)	Error	Time(s)	Error
7	0.007	**1.60e−07**	0.004	3.18e−07	0.002	3.17e−05	0.001	2.93e−07
10	0.019	1.05e−09	0.004	5.71e−07	0.004	5.03e−10	0.004	**3.70e−10**
13	0.019	1.05e−09	0.069	5.24e−07	0.017	8.04e−09	0.022	**3.11e−10**

Significant values are in bold.

**Table 14:** Comparision of maximum relative error of $z_3(t)$ for Example 7.3 with different numbers of neurons.

n	Legendre		Laguerre		Hermite		Chebyshev	
	Time(s)	Error	Time(s)	Error	Time(s)	Error	Time(s)	Error
7	0.007	**1.31e−07**	0.004	1.76e−07	0.002	1.78e−07	0.001	1.74e−07
10	0.019	2.82e−08	0.004	4.05e−07	0.004	1.27e−08	0.004	**3.91e−09**
13	0.019	5.18e−08	0.069	5.65e−07	0.017	4.23e−08	0.022	**5.74e−09**

Significant values are in bold.

## Conclusion

In this paper, we obtained approximate solutions of higher order NDDEs, as well as a system of DDEs with multiple delays and variable coefficients, using four single-layer orthogonal polynomial-based neural networks: (i) Legendre neural network, (ii) Chebyshev neural network, (iii) Hermite neural network, and (iv) Laguerre neural network. For training the network weights, the ELM algorithm is used. It is proved that the relative error between the exact solution and approximate solutions obtained by ONNs is  of order $2^{-n}$, where $n$ is the number of neurons. Further, it is shown that each orthogonal polynomial-based neural networks provide an approximate solution, that are in good agreement with the exact solution. However, it is observed that, among these four ONNs, the Chebyshev neural network provides the most accurate result.

The results in the section (6), (7) demonstrate that the proposed method is simple to implement and a powerful mathematical technique for obtaining the approximate solution of the higher order NDDEs as well as the system of DDEs.

## Data Availability

The data that support the findings of this investigation are accessible from the authors upon reasonable request. If necessary, you can contact by email sdubey@iitm.ac.in.
